# Identifying and validating blood mRNA biomarkers for acute and chronic insufficient sleep in humans: a machine learning approach

**DOI:** 10.1093/sleep/zsy186

**Published:** 2018-09-24

**Authors:** Emma E Laing, Carla S Möller-Levet, Derk-Jan Dijk, Simon N Archer

**Affiliations:** 1 Department of Microbial Sciences, School of Biosciences and Medicine, Faculty of Health and Medical Sciences, University of Surrey, Guildford, UK; 2 Bioinformatics Core Facility, Faculty of Health and Medical Sciences, University of Surrey, Guildford, UK; 3 Surrey Sleep Research Centre, School of Biosciences and Medicine, Faculty of Health and Medical Sciences, University of Surrey, Guildford, UK

**Keywords:** sleep deprivation, biomarkers, blood, transcriptomics, elastic net, regression, multivariate

## Abstract

Acute and chronic insufficient sleep are associated with adverse health outcomes and risk of accidents. There is therefore a need for biomarkers to monitor sleep debt status. None are currently available. We applied elastic net and ridge regression to transcriptome samples collected in 36 healthy young adults during acute total sleep deprivation and following 1 week of either chronic insufficient (<6 hr) or sufficient sleep (~8.6 hr) to identify panels of mRNA biomarkers of sleep debt status. The size of identified panels ranged from 9 to 74 biomarkers. Panel performance, assessed by leave-one-subject-out cross-validation and independent validation, varied between sleep debt conditions. Using between-subject assessments based on one blood sample, the accuracy of classifying “acute sleep loss” was 92%, but only 57% for classifying “chronic sleep insufficiency.” A reasonable accuracy for classifying “chronic sleep insufficiency” could only be achieved by a within-subject comparison of blood samples. Biomarkers for sleep debt status showed little overlap with previously identified biomarkers for circadian phase. Biomarkers for acute and chronic sleep loss also showed little overlap but were associated with common functions related to the cellular stress response, such as heat shock protein activity, the unfolded protein response, protein ubiquitination and endoplasmic reticulum-associated protein degradation, and apoptosis. This characteristic response of whole blood to sleep loss can further aid our understanding of how sleep insufficiencies negatively affect health. Further development of these novel biomarkers for research and clinical practice requires validation in other protocols and age groups.

**Statement of Significance**

Insufficient sleep poses a significant risk to physical and mental health, and the general safety of individuals. The development of an objective assessment is important for treatment and diagnosis, with applications in many real world situations, for example, suspected sleepy (drowsy) driver accidents, assessing “fitness for duty,” interpretation and diagnosis of sleep disorders, and evaluation of therapeutic sleep interventions. Here, we show that sets of blood mRNA transcripts, related to biological functions associated with cellular stress responses, can accurately predict acute sleep loss but detecting chronic sleep insufficiency is more challenging.

## Introduction

A multitude of recent epidemiological and interventional studies have established that insufficient sleep is associated with adverse health outcomes. Excessive sleepiness, impaired sustained attention, changes in mood, and a variety of adverse physical health outcomes such as obesity, diabetes, and cardiovascular disease, have all been demonstrated to be linked to insufficient sleep [1–4]. Indeed, it has been recommended that adults sleep 7 or more hours per night to avoid the adverse health outcomes associated with sleep debt [5]. However, in most studies quantification of sleep debt does not extend beyond subjective assessment. Objective assessment of sleep debt status will aid the development of the next generation of epidemiological studies on the health consequences of insufficient sleep. Objective assessment of sleep debt status also has direct applications in many real world situations, for example, suspected sleepy (drowsy) driver accidents, assessing “fitness for duty,” diagnosis of sleep disorders, and evaluation of therapeutic sleep interventions [4, 6, 7]. Yet, methods that can objectively assess sleep debt status and be deployed on a large scale are currently not available.

Experimental research approaches to identify putative biomarkers for sleep debt status typically include laboratory studies of acute sleep deprivation and chronic sleep restriction. Acute total sleep deprivation consists of foregoing sleep and being awake for more than 24 hr, whereas chronic sleep restriction can, for research purposes, be defined as getting some but insufficient (e.g. <6 hr) sleep during consecutive (e.g. 3 or more) 24 hr periods [6]. Biomarker discovery can make use of univariate and multivariate high-throughput data derived from samples (blood, saliva, urine, or specific tissues and/or organs [6, 8]) collected in these protocols. The transcriptomes of organs, such as the brain and liver, change in response to acute total sleep deprivation, and sleep displacement and disruption, as has been shown in animal studies [9–12]. In humans, biomarker discovery has focused on samples from blood, saliva, or urine, because internal organs are not easily accessible. Early attempts to identify biomarkers for sleep debt in easily accessible sources such as saliva, were informed by transcriptomic studies, with one particular molecule, amylase, identified as a putative biomarker for acute total sleep loss in drosophila and humans. Levels of mRNA encoding amylase and amylase enzymatic activity were both elevated in the saliva of individuals who experienced acute total sleep deprivation [13]. Metabolomic approaches have identified changes in blood metabolites in response to chronic sleep restriction in rodents and humans. In a carefully controlled study, the molecules oxalic acid and diacylglycerol 36:3 were identified as putative cross-species biomarkers of chronic sleep debt [14]. However, these pioneering studies neither quantified nor independently validated the ability of the putative biomarkers to monitor sleep debt at the level of the individual. Furthermore, these studies focused on univariate biomarkers and no attempts were made to identify a panel. A panel consisting of multiple features is likely to be more sensitive and robust than an assessment based on a single feature [8]. Multivariate approaches have been shown to be successful in a number of areas including biomarkers for cancer and neurodegeneration [8].

Theoretical models predict [15], and experimental sleep studies have shown [16], that in humans sleep propensity is governed by two processes, sleep homeostasis and circadian rhythmicity. Identification of biomarkers of the status of these processes is pertinent to our understanding of sleep regulation. Recently, we have demonstrated that the blood transcriptome in one or two blood samples contains sufficient information to reliably predict the circadian phase of the master pacemaker in the human hypothalamus as indexed by the plasma melatonin rhythm [17]. Here, we investigate whether a panel of blood transcriptome features can be used to predict the homeostatic aspect of sleep regulation, that is, sleep debt status.

The human blood transcriptome is affected by acute total sleep deprivation and chronic sleep restriction, as well as sleep timing and circadian phase [18–21]. This implies that the blood transcriptome is a potential source for identifying biomarkers associated with sleep debt variables with the provision that confounding factors such as circadian phase are adequately considered and taken into account. Our previously reported effects of total sleep deprivation and chronic sleep restriction [18] were based on time series of 10 transcriptome samples collected across the circadian cycle in each participant and in two sleep debt conditions. Significant effects of sleep debt condition were assessed for individual transcripts at the group level, using a within participant comparison. Ideally a biomarker should not require many samples or a within participant comparison. Accordingly, here, we compare approaches to identify and validate multivariate blood transcriptome biomarker panels for assessing the acute and chronic sleep debt status of an individual by using only a few samples, and compare the molecular characteristics of the panels.

Identifying a panel of mRNA biomarkers for a given condition of interest, for example, chronic insufficient sleep, represents a feature selection problem. There are different approaches to selecting features from large scale data; univariate filtering (i.e. application of prior knowledge, statistical, and/or correlation thresholds), multivariate wrapped (e.g. partial least-squares regression [17]), and/or embedded methods (e.g. LASSO [22] and elastic net [23] penalized regression techniques). We have previously shown that *a priori* knowledge-based filtering may not be the optimal approach for developing a predictive model of circadian phase [17]. Nevertheless, prefiltering of features, before the application of multivariate wrapped/embedded methods, has been reported to be advantageous in some situations [24]. Here, we have therefore compared these different approaches in identifying biomarkers for sleep debt status. Validation is essential in evaluating biomarker performance. Although independent validation (IV) is the gold standard, validation methods such as leave-one-out or k-fold cross-validation that assess performance by resampling the training set have been developed to overcome the constraints of limited data [25, 26]. Thus, we also compared cross-validation and IV, as well as different approaches to partitioning samples to create training and validation sets. In addition, we assessed whether the sleep debt status of an individual can be assessed without any prior knowledge about the individual (between subject assessments) or whether prior knowledge (within subject assessment) is required.

## Methods

### Source of data and definitions of various indices of sleep debt status

**Figure 1.**
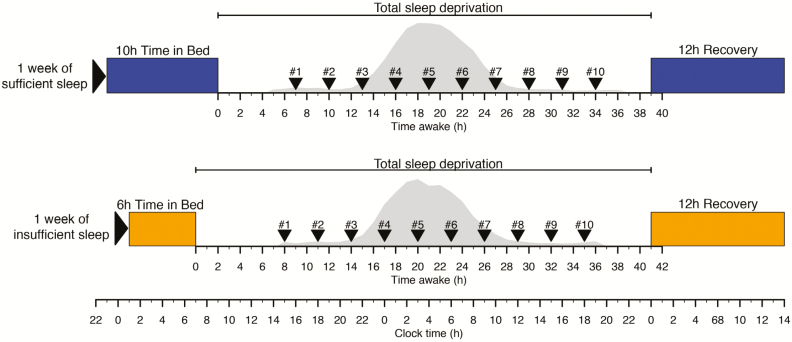
Protocol. In a crossover design participants entered a constant routine following either 1 week of sufficient (mean ± SEM of PSG assessed TST of 8.56 ± 0.06 hr) sleep or 1 week of insufficient (mean ± SEM of PSG assessed TST of 5.75 ± 0.06 hr) sleep. During the constant routine of 39–41 hr of wakefulness blood samples were collected 3 hourly for transcriptome samples (samples labeled #1 to #10) and hourly for melatonin assessments. At the end of each constant routine participants were given a 12 hr recovery period. Melatonin curves (gray area) represent the average melatonin curve (across all participants) during the constant routine for sufficient and insufficient sleep, respectively.

**Figure 2.**
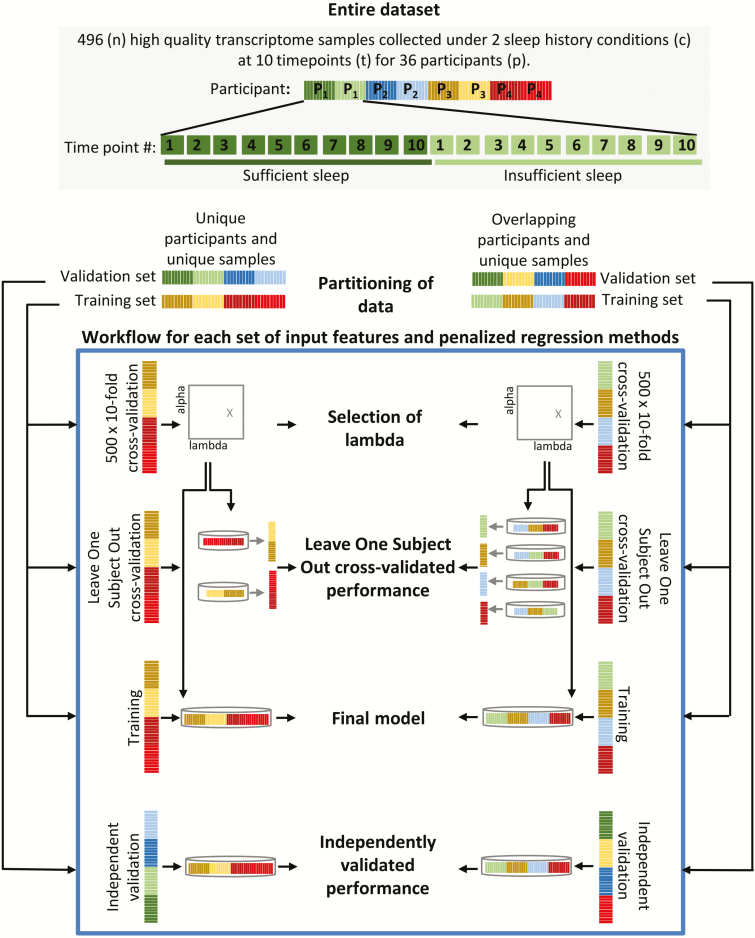
Overview of our approach to identifying panels of biomarkers and assessing their performance.

Data were collected in a previously described study that received a favorable opinion from the University of Surrey’s Ethics committee in accordance with the guidelines laid down in the Declaration of Helsinki. All participants provided written informed consent [18]. The chronic sleep debt status of 36 (18 males; mean ± SD of age = 27.6 ± 4.0 years) healthy participants without sleep disorders and having an average actigraphically assessed habitual time in bed (TIB) of 8 hr and 18 min, was manipulated by enforcing 1 week of insufficient sleep (6 hr TIB, mean ± SEM of polysomnography [PSG] assessed total sleep time [TST] of 5.75 ± 0.06 hr) and sufficient sleep (10 hr TIB, mean ± SEM of PSG assessed TST of 8.56 ± 0.06 hr) under carefully controlled clinical research center conditions in a balanced crossover design (see Figure 1 and [2]). In these same participants, acute sleep debt status was manipulated by enforcing a 39–41 hr period of wakefulness (total sleep deprivation), under constant routine conditions, immediately following the week of sufficient and insufficient “sleep history” condition. In the constant routine condition, participants were in a semi-recumbent position under constant dim light. Their caloric needs were met by hourly “nutritional beverages.” Three-hourly (nonfasting) whole-blood RNA samples, labeled samples #1 to #10, were collected through an indwelling cannula in the participant’s forearm (without heparin) during each of these total sleep deprivation periods from each participant. The first sample (#1) was taken at approximately 15:00–16:00 hr, that is, 7–8 hr after scheduled wake time, and sample #10 at approximately 18:00–19:00 hr, that is, 34–35 hr after scheduled wake time. These data represent an extension of the data previously analyzed [18].

Based on this data set, we explored the feasibility of biomarker development for the variables:

Time awake, the number of hours awake, given a blood sample taken at any time of day.
Wakefulness of more than 24 hr, given a blood sample taken at any time of day.
Acute sleep loss (skipped a night of sleep), given a blood sample taken in the afternoon.
Chronic sleep insufficiency, given a blood sample taken in the afternoon.
Sleep increase/decrease, given two blood samples of an individual taken on different occasions.

Ideally a biomarker should be able to identify the status of an individual by assessing the value of the biomarker without any information about the value of the biomarker in that individual in another state, that is, the value of the biomarker can be compared with the population distribution to assess status. In cases in which the subject-specific (“trait” like) variation in the value of the biomarker is large compared with the “state” like variation, status can only be assessed by assessing changes in the biomarker within an individual. For example, the notion that slow-wave sleep is a biomarker of sleep homeostasis is largely based on within-subject changes and the between-subject variation, which is large, is ignored. Likewise, the human transcriptome is highly individual [27] and this poses a challenge for the development of biomarkers that can predict the sleep debt status of individuals by a between-subject comparison. A between-subject approach is nevertheless preferable. We have therefore compared biomarkers which can be used for a between-subject comparison (based on single, independently collected transcriptome samples; predictor algorithms are blind to the subject from whom a sample is taken) and biomarkers which can be used for a within-subject assessment of sleep debt status, or more precisely, assessment of a change in sleep debt status.

### Creation of training and validation data sets

Following quality control assessments (i.e. sufficient quality and quantity of RNA), at least one whole-blood transcriptome sample was obtained for 36 participants, with a total of 496 transcriptome samples across the study (254 following 1 week of sufficient sleep, 242 following 1 week of insufficient sleep). All raw data are accessible from the Gene Expression Omnibus [28] (Accession numbers: GSE39445 and GSE82114).

Two different approaches were taken to create pairs of training and validation sets from the 496 samples (Figure 2); (1) “unique participants and unique samples” (UPUS), where all samples collected from a single participant were included in either the training or validation set, and the training and validation sets each comprise a unique set of participants; (2) “overlapping participants and unique samples” (OPUS), where all samples collected from a participant during one sleep history condition (sufficient or insufficient sleep) were included in the training set, and all samples collected during the alternate sleep history condition were included in the validation set. In this split, all participants are present in both the training and validation sets, and it is only the samples that are unique between sets (with sleep history conditions balanced within each set). Partitioning the entire data set by these two approaches was conducted randomly while ensuring that relevant participant characteristics such as gender were balanced across the training and validation sets. The structure of the training and validation sets remained the same across all analyses except for the sleep variable “sleep increase/decrease,” where an additional factor “clinic visit” was required to be balanced. Where analyses rely on a subset of samples the nonrelevant samples were discarded from the training and validation sets. The processed data sets are available at http://sleep-sysbio.fhms.surrey.ac.uk/SleepDebt.

### Data processing

The log_2_ mRNA abundance values within each sample were 75th percentile normalized. Noncontrol technically replicated probes (mRNA abundance features), along with their corresponding Agilent feature flags (Agilent Feature Extraction Software [v10.7], Reference Guide, publication number G4460-90036) were averaged. Following standard operating procedures, features (probes) flagged as poor quality based on AgilentQC metrics of reproducibility statistics, minimum detection level estimates, and feature flags (Agilent Feature Extraction Software [v10.7], Reference Guide, publication number G4460-90036) in more than 10% of the samples within the training set were removed. Thus, each training-validation data set pair were distinct in the number of samples, participants, and probes that they contain. For within-subject assessments, each sample within a data set was transformed to reflect the change in mRNA abundance between the reference and test sample (e.g. sample #2 − sample #1 [baseline]). This procedure removes the trait-like (i.e. participant specific) characteristics of the blood transcriptome and thereby may facilitate the changes in the transcriptome associated with the sleep intervention.

### Feature selection

As each processed transcriptome sample comprises ~20 k mRNA abundance features (probes), with one or more features targeting transcripts of a single gene, we applied different approaches to select features. Approaches involved the use of *a priori* knowledge (i.e. using prefiltered features) and the use of penalized (regularized) linear regression techniques with and without prefiltered features.

Regularization is a method by which the complexity of a model can be controlled through the use of penalties. Penalties referred to as L1 norm and L2 norm are used to control the least absolute error and/or least squares error between the predicted and observed target value, respectively. High L1 norm penalties, such as applied in LASSO regression [22], produce sparse, low complexity models. A reduced subset of all features is selected to predict the desired outcome, where the selection of a “representative” feature(s) from a set of highly correlated features is arbitrary. Conversely, L2 norm penalties, such as applied in ridge regression [29], produce more complex models comprising all features (no selection) with small distributed weights. Alternative, “hybrid” methods, such as elastic net regression [23], combine the L1 and L2 penalties to generate sparse models (as per LASSO) whilst allowing for all correlated features, such as those targeting transcripts encoding proteins involved in the same pathway/biological process, to appear in the model (as per ridge) [23]. For studies aiming to identify putative blood based biomarkers, and not just develop a prediction/classification model, elastic net has shown great promise [30].

#### Feature selection using a priori knowledge

It may be argued that a biomarker for acute total sleep loss should neither be influenced by the circadian phase at which a sample is taken, nor the prior sleep history. Thus, in our prefiltering approach features were selected *a priori* based on their own independent mRNA abundance profiles across time, from our within-subject analyses of a subset of the current data reported in Möller-Levet et al. [18]. Features selected *a priori* for models associated with hours awake, herein referred to as the “hours awake” *a priori* selected set of features, met the criteria that: (1) their mRNA abundance profiles displayed a significant positive/negative trend across time following both sufficient and insufficient sleep, (2) their profiles did not display a significant circadian modulation during the time awake period, and (3) their profiles were not significantly different between sleep history conditions. Features selected *a priori* for models associated with predicting the sufficiency of sleep (e.g. sleep history of 10 hr TIB vs. 6 hr TIB), herein referred to as the “sleep sufficiency” *a priori* selected set of features, met the criteria that: (1) their mRNA abundance profiles displayed significant differences between sleep history conditions, (2) their profiles did not display a significant circadian modulation during the time awake period, and (3) their profiles did not display a significant positive or negative trend across time, that is, were not affected by acute sleep loss. Prediction/classification models generated from features selected by *a priori* knowledge were developed using ridge regression. Due to the independent data processing applied not all of the *a priori* selected features may appear within a given training set. Table 1 details the number of *a priori* selected features available for training.

#### Feature selection using elastic net regression

Elastic net regression was used to concurrently select features and produce a linear regression model for predicting/classifying a given sleep debt variable using: (1) all features (no *a priori* knowledge), or (2) an *a priori* selected set of features, as input.

There are two tuning hyper-parameters that influence feature selection and model construction in penalized regression approaches such as elastic net regression: alpha, which controls the balancing of L1 norm and L2 norm penalties, and lambda, which controls the level of the penalty(s) given alpha. Here, alpha was set to 0.5 for elastic net regression and 0 for ridge regression. To determine the value of lambda, we performed a 10-fold cross-validation search for the lambda value that gives the minimum mean cross-validated error when applied to the given training set. Folds of the training set were assigned randomly, albeit with respect to the balance of classes within the training set, and thus repeated 500 times. The mean value of the selected lambda across the 500 searches was subsequently defined as the “optimal” lambda value for creating the final prediction/classification model based on all samples within the training set. Features with nonzero coefficients within the final model were defined as biomarkers relevant for predicting/classifying the target value.

The performance of the final model (panel of biomarkers) was assessed using (1) leave-one-subject-out cross-validation (LOSO-CV) of the training set (a different strategy to the cross-validation approach applied when selecting lambda), and (2) IV using the corresponding independent validation test set. Comparing LOSO-CV and IV performance we were able to identify any “overfitting” of the model to the training set, a common problem when using small data sets [26]. All models were developed and tested using the glmnet package (version 2.0–13) [31] in R (version 3.3.3) [32].

### Measuring model performance

Prediction models used to predict a quantitative target value $\hat{y}$ (e.g. number of hours awake) were linear regression models of the form:

$$\;\hat{y}=\beta_{0}+\beta_{1}x_{1}+\ldots \beta_{n}x_{n}$$

where *x* are all features provided as input, β the coefficients assigned to each feature by the penalized regression approach, and the intercept β_0_. All input features appear in the model. Features not selected by penalized regression were assigned a coefficient of 0. Performance of such models was assessed by *R*^2^, a measure of the fit between the predicted ${\hat{y}}_{i}$ and observed *y*_*i*_ target value for each of the transcriptome samples comprising *x* features within the test set:

$$R^{2}=1-\frac{\sum_{i=1}^{n}{\left(y_{i}-{\hat{y}}_{i}\right)}^{2}}{\sum_{i=1}^{n}{\left(y_{i}-{\bar{y}}_{i}\right)}^{2}}$$

Classification (logistic regression) models used to classify a sample/individual into one of two classes were of the form:

$$\hat{y}=\frac{e^{\beta_{0}+\beta_{1}x_{1}\ldots \beta_{n}x_{n}}}{1+e^{\beta_{0}+\beta_{1}x_{1}\ldots \beta_{n}x_{n}}}$$

where *x* are all features provided as input, β the coefficients assigned to each feature by penalized regression, and the intercept β_0_. All features appear in the model, with only selected features having a nonzero coefficient. Here, $\hat{y}$ represents the probability that a participant with a given mRNA abundance value for features *x* belongs to a given class. The use of the term ${\text{e}}^{\beta_{0}+\beta_{1}x_{1}...\beta_{n}x_{n}}$ in both the numerator and denominator ensures that output value $\hat{y}$will fall between 0 and 1. As samples belonging to each class were balanced as much as possible within the training and validation sets, the prior probabilities for each class were 0.5. Thus, the decision boundary of 0.5 was used to assign a transcriptome sample to a particular class.

For classification the target class variable (e.g. “chronic sleep insufficiency,” or “acute sleep loss”) was considered to be the “positive” class, and the alternate class (e.g. “no chronic sleep insufficiency” or “no acute sleep loss”) was considered to be the “negative” class. Subsequently, we were able to determine the number of true positives (TP), true negatives (TN), false positives (FP), and false negatives (FN). The performance of a classification model was assessed by overall accuracy [TP + TN/(TP + TN + FP + FN)], sensitivity [TP/(TP + FN)], specificity [TN/(TN + FP)], and Matthew’s correlation coefficient [MCC:$\text{TP.TN-FP.FN}/\sqrt{\text{(TP+FP)(TP+FN)(TN+FP)(TN+FN)}}$], a consistent measure of model accuracy invariant to the balancing of samples [33].

Figures for visualization of model performance, and features’ mRNA abundance profiles were created in R (version 3.3.3) [32] using packages: heatmap3 (version 1.1.1) [34], gplots (version 3.0.1) [35], and Hmisc (version 4.1-1) [36].

### Calculating effect size

Cohen’s *d* effect size, the difference in the mean predicted value *M* between two groups in pooled (across the two groups) *SD* (*s*) units, was calculated by:

$$d=\frac{M_{1\;\;}-M_{2\;}}{s_{\text{pooled}}}$$

Groupings were based on the observed value for the variable “time awake, between-subject.”

### Functional analysis and interpretation of panels of biomarkers

Each panel of biomarkers was transformed to a list of unique gene symbols (indicated in Table 1). Statistical functional enrichment analysis was performed on each gene list using the online tool Metascape [37] at www.metascape.org (based on annotation update on January 1, 2018) with default settings and the human protein-encoding genome as the background. Information on gene function/interactions was sourced from the GeneCards database [38] at www.genecards.org (v4.7.2) and UniProt [39] at www.uniprot.org (release 2018_03). The full list of gene ontology (GO) annotations for all gene symbols was downloaded from UniProt [39] at www.uniprot.org (release 2018_03). This annotation was used to identify the “top 10” enriched GO terms for each panel of biomarkers, where enrichment is based on the proportion of genes in the list associated with a GO term (no statistical test applied). Known and inferred functional interactions between genes were extracted from the STRING database [40] at string-db.org (v10.5) using default settings. Briefly, confidence in the association (functional interaction) of a pair of genes was calculated by combining several sources of evidence, including experimentally observed interactions in human and/or other species, inference based on text-mining and conservation across multiple species. A pair of genes having a combined confidence score of at least 0.4, which excludes low quality confidence scores, were considered to be associated and thus appear connected by an edge in the resultant functional interaction network. Redrawing of STRING inferred networks and network analysis (degrees for each node in the network) was conducted using Cytoscape (version 3.2.1 [41]). All connected nodes were included.

**Table 1.** Size and performance of biomarker panels for the prediction/classification of different sleep debt variables

Biomarkers for		Number of samples within the training and independent validation sets	Number of features (unique genes) in the final model			LOSO-CV performance			IV performance		
			*a priori* knowledge and ridge	*a priori* knowledge and elastic net	All features and elastic net	*a priori* knowledge and ridge	*a priori* knowledge and elastic net	All features and elastic net	*a priori* knowledge and ridge	*a priori* knowledge and elastic net	All features and elastic net
Acute sleep loss variables	Prediction of “time awake, between-subject”; samples #1 to #10	Training = 239 Validation = 233	26 (26)	17 (17)	59 (58)	*R* ^2^ = 0.31	*R* ^2^ = 0.30	*R* ^2^ = 0.35	*R* ^2^ = 0.23	*R* ^2^ = 0.22	*R* ^2^ = 0.29
	Prediction of “time awake, within-subject”; sample #2 - sample #1, #3 - #1, #4 - #1, #5 - #1, #6 - #1, #7 - #1, #8 - #1, #9 - #1, #10 - #1	Training = 185 Validation = 189	26 (26)	9 (9)	74 (73)	*R* ^2^ = 0.23	*R* ^2^ = 0.25	*R* ^2^ = 0.44	*R* ^2^ = 0.22	*R* ^2^ = 0.24	*R* ^2^ = 0.30
	Classification of “wakefulness of more than 24 hr, between-subject”; all samples, based on the predicted “time awake” value	Training = 239 Validation = 233	26 (26)	17 (17)	59 (58)	ACC = 75% Sn = 61% Sp = 84% MCC = 0.47	ACC = 74% Sn = 61% Sp = 84% MCC = 0.46	ACC = 79% Sn = 66% Sp = 89% MCC = 0.57	ACC = 74% Sn = 50% Sp = 91% MCC = 0.46	ACC = 73% Sn = 49% Sp = 88% MCC = 0.42	ACC = 80% Sn = 70% Sp = 87% MCC = 0.59
	Classification of “acute sleep loss, between-subject”; samples #1 vs. sample #9	Training = 50 Validation = 49	26 (26)	20 (20)	68 (68)	ACC = 76% Sn = 71% Sp = 79% MCC = 0.51	ACC = 72% Sn = 76% Sp = 69% MCC = 0.45	ACC = 76% Sn = 76% Sp = 76% MCC = 0.52	ACC = 76% Sn = 52% Sp = 93% MCC = 0.51	ACC = 76% Sn = 57% Sp = 89% MCC = 0.5	ACC = 92% Sn = 90% Sp = 93% MCC = 0.83
	Classification of “acute sleep loss, within- subject”; difference between sample #2 and #1 vs. difference between sample #10 and #1	Training = 41 Validation = 43	26 (26)	12 (12)	32 (31)	ACC = 88% Sn = 87% Sp = 89% MCC = 75%	ACC = 93% Sn = 87% Sp = 100% MCC = 0.86	ACC = 90% Sn = 83% Sp = 100% MCC = 0.82	ACC = 77% Sn = 72% Sp = 83% MCC = 0.55	ACC = 74% Sn = 68% Sp = 83% MCC = 0.51	ACC = 74% Sn = 72% Sp = 78% MCC = 0.49
Chronic sleep loss variables	Classification of “chronic sleep insufficiency”; samples #1 or #2 for insufficient sleep vs. samples #1 or #2 for sufficient sleep	Training = 30 Validation = 30	407 (407)	14 (14)	9(9)	ACC = 60% Sn = 67% Sp = 53% MCC = 0.2	ACC = 53% Sn = 53% Sp = 53% MCC = 0.07	ACC = 53% Sn = 60% Sp = 47% MCC = 0.07	ACC = 57% Sn = 62% Sp = 53% MCC = 0.14	ACC = 47% Sn = 54% Sp = 41% MCC = -0.05	ACC = 50% Sn = 77% Sp = 29% MCC = 0.07
	Classification of “chronic sleep insufficiency”; samples #9 or #10 for insufficient sleep vs. samples #9 or #10 for sufficient sleep	Training = 34 Validation = 30	420 (420)	7 (7)	21 (21)	ACC = 59% Sn = 65% Sp = 53% MCC = 0.18	ACC = 59% Sn = 71% Sp = 47% MCC = 0.18	ACC = 47% Sn = 47% Sp = 47% MCC = -0.06	ACC = 53% Sn = 38% Sp = 71% MCC = 0.09	ACC = 57% Sn = 50% Sp = 64% MCC = 0.14	ACC = 47% Sn = 44% Sp = 50% MCC = -0.06
	Classification of “sleep decrease/increase”; difference in sample #2 or sample #3 from visit 1 and sample #2 or sample #3 from visit 2	Training = 10 Validation = 9	413 (413)	13 (13)	36 (34)	ACC = 70% Sn = 80% Sp = 60% MCC = 0.41	ACC = 50% Sn = 60% Sp = 40% MCC = 0	ACC = 50% Sn = 60% Sp = 40% MCC = 0	ACC = 89% Sn = 100% Sp = 80% MCC = 0.8	ACC = 78% Sn = 100% Sp = 60% MCC = 0.63	ACC = 78% Sn = 100% Sp = 60% MCC = 0.63
	Classification of “sleep decrease/increase”; difference in sample #9 or sample #10 from visit 1 and sample #9 or sample #10 from visit 2	Training = 14 Validation = 15	413 (413)	18 (18)	62 (62)	ACC = 64% Sn = 57% Sp = 71% MCC = 0.29	ACC = 71 % Sn = 71% Sp = 71% MCC = 0.43	ACC = 71% Sn = 71% Sp = 71% MCC = 0.43	ACC = 67% Sn = 100% Sp = 44% MCC = 0.49	ACC = 53% Sn =83 % Sp = 33% MCC = 0.18	ACC = 40% Sn = 83% Sp = 11% MCC = -0.08

Data shown for models based on “UPUS” training and validation sets only.

ACC = classification accuracy, Sn = sensitivity, Sp = specificity, MCC = Matthew’s correlation coefficient.

## Results

### Biomarker performance

Table 1 lists the number of features and unique genes, and the corresponding LOSO-CV and IV performance for all panels of biomarkers identified and validated using the UPUS data sets. Supplementary Table S1 provides the same information for panels of biomarkers identified and validated using OPUS data. Performance plots not shown and discussed herein (e.g. performance of all *a priori* knowledge informed panels of biomarkers, and panels identified using OPUS data) are provided in Supplementary File S1.

#### Biomarker panels for acute sleep loss

##### Predicting “time awake”; between-subject assessment.

We first investigated whether it is possible to predict the time an individual has been awake from one blood transcriptome sample. Every sample was assigned a time awake value ranging from 7 to 35 hr (Figure 1). Training and corresponding validation sets included all samples and were submitted to the workflow (Figure 2) to identify and validate biomarker panels for “time awake” using different approaches.

**Figure 3.**
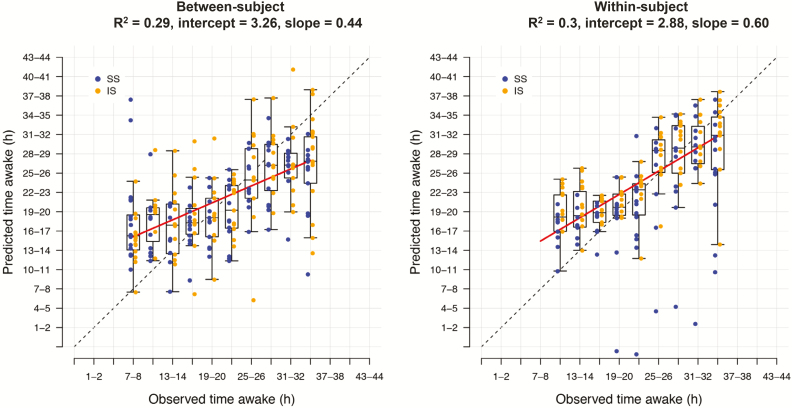
Prediction performance for “time awake” between- and within-subject. Prediction of “time awake” from one (between-subject) or two (within-subject) transcriptome samples. Blue: samples taken following 1 week of sufficient sleep; gold: samples taken following 1 wk of insufficient sleep. Dashed line indicates the line of unity. Red line indicates a linear regression of the relationship of predicted and observed. Data shown is based on the final “all features” elastic net model trained on all samples within the “UPUS” training set and applied to all samples within the corresponding independent validation set.

The panels of biomarkers identified as having the best performance, highest *R*^2^ value, were obtained when using “all features” and elastic net (UPUS *R*^2^ = 0.29, OPUS *R*^2^ = 0.39). These panels comprised 59 (UPUS) and 211 (OPUS) features. The LOSO-CV and IV performance of panels identified and validated from the training and validation sets were similar, although IV performance was somewhat lower than LOSO-CV (Table 1, Supplementary Table S1). Panels identified and validated using OPUS data yielded higher performance (*R*^2^ range between 0.23 and 0.31 for UPUS, 0.28 and 0.45 for OPUS). However, the *R*^2^ values obtained from all searches is considered poor, with less than 50% of the observed variance in the values of “time awake” explained (Figure 3a).

##### Predicting “time awake”; within-subject assessment.

Our previous analyses of a subset of the current data identified 22,401 features for which there is a significant effect of time on mRNA abundance *within* a participant [18]. Our above approach to predicting “time awake” does not consider the individual. Here, we further processed all mRNA abundance values to reflect change from the participant’s baseline (sample #1). Each baseline-corrected transcriptome sample was assigned the same time awake value as the nonbaseline corrected sample and used to identify biomarkers for “time awake” within an individual. Baseline correction (within participant comparison) did not improve prediction performance and all R^*2*^ values were weak (R^*2*^ range of 0.15–0.44 across approaches) and similar to that observed when predicting “time awake” without considering the individual (Figure 3). The “all features” elastic net approach again identified panels with the highest performance. However, the panels from the two-sample approach were of different size to those identified by the one-sample approach (74 UPUS, 184 OPUS).

##### Classifying wakefulness of more than 24 hr; between-subject assessment.

**Figure 4.**
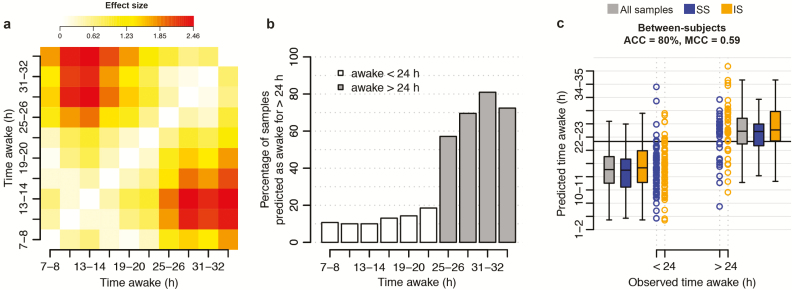
Predicted “time awake” value for classifying “wakefulness of >24 hr.” (a) Difference between the mean predicted value of “time awake” for samples collected at *x* hours awake and samples collected at *y* hours awake. Difference expressed as Cohen’s *d* effect size. Data based on predictions made for samples within the “UPUS” training data set when using the “all features” elastic net model for “time awake” trained on all samples within the “UPUS” training data set. No baseline correction applied. (b) Percentage of samples predicted to have a “time awake” value of greater than 24 hr for all observed values of “time awake.” Data based on predictions made for samples within the “UPUS” validation data set when using the “all features” elastic net model for “time awake” trained on all samples within the “UPUS” training data set. (c) Classification performance when classifying a sample within the “UPUS” validation set to one of two classes, “awake > 24 hr,” “awake < 24 hr” using the predicted “time awake” value of a sample using the “all features” elastic net model for “time awake” trained on all samples within the “UPUS” training data set. Black horizontal line represents the decision boundary at 24 hr. ACC = accuracy, SS= samples from sufficient sleep condition, IS = samples from insufficient sleep condition.

A second question is whether we can reliably predict whether an individual has been awake for more than 24 hr or not. While the *R*^2^ values between predicted and observed “time awake” values were poor, the panels clearly predict a trend in time awake. The average predicted value for a given observed value of “time awake” was typically higher than the average predicted value for the preceding values of “time awake” (Figure 3). Indeed, the effect sizes between the predicted values for each observed “time awake, between-subject” value pair were greater than 0.2 *SD*s (effect size *d* > 0.2; Figure 4a). Large (*d* > 0.8) effect sizes were observed when comparing the mean predicted “time awake, between-subject” values for sampling points #1 to #6 (awake less than 24 hr) with that of samples #7 to #10 (awake more than 24 hr). This breaking point in effect size suggested that the predicted “time awake” values may be used to classify a sample. Thus, for every observed “time awake, between-subject” value, we assessed the percentage of predicted values having a value greater than 24 hr. As expected, the distributions of these percentage values were distinct for observed “time awake, between-subject” values of <24 hr and >24 hr (Figure 4b). Subsequently, for a given panel of biomarkers, we used a sample’s predicted “time awake, between-subject” value to assign it to one of two classes; “awake < 24 hr” or “awake > 24 hr,” with “awake > 24 hr” considered to be the “positive” class.

The “all features” and elastic net approach was again found to produce the greatest performance with an overall classification accuracy of 79% (OPUS) to 80% (UPUS) (Table 1, Figure 4c). All panels of biomarkers were more specific (specificity: 84%–91%) in their classification than sensitive (sensitivity: 49%–70%).

##### Classifying “acute sleep loss” without confounding by circadian phase; between-subject assessment.

In all analyses presented thus far, circadian phase and time awake changed simultaneously. We next addressed whether it is possible to determine if a sample taken at a particular circadian phase comes from an individual who has been awake during the preceding 24 hr (i.e. skipped one night of sleep). Indexing samples by clock time, each sample was assigned to either class of “acute sleep loss” or “no acute sleep loss” by the presence/absence of sleep within the same 24 hr clock period. Samples #1 and #9 from both sleep history conditions were retained because both samples were collected at the same time of day without the confounding effect of circadian phase and represent negative and positive cases of “acute sleep loss,” respectively.

**Figure 5.**
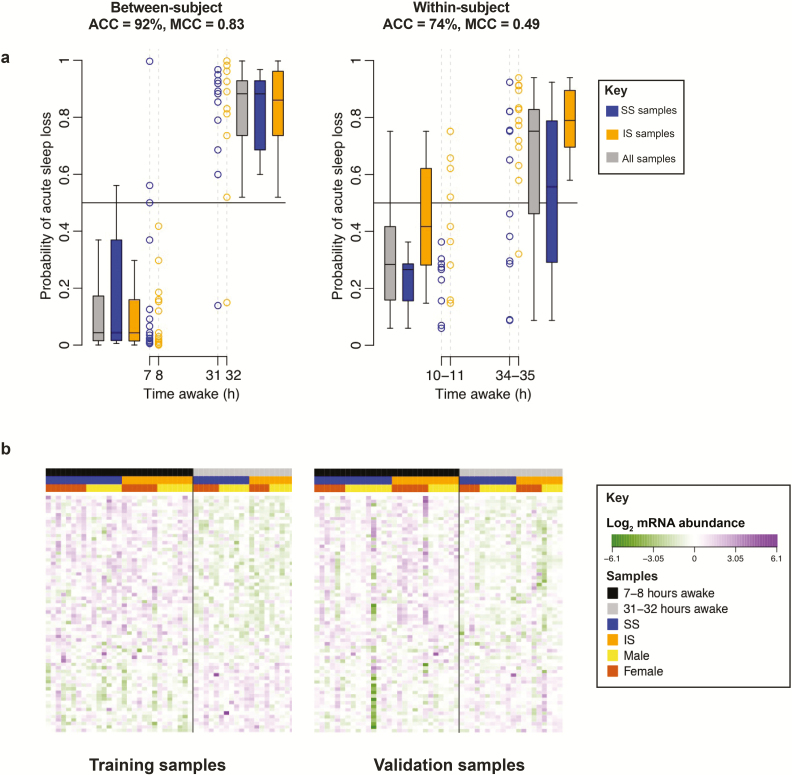
Classification performance for “acute sleep loss” between- and within-subject. (a) Classification of a sample to two classes: “acute sleep loss” and “no acute sleep loss.” “Between-subject” refers to the classification of “acute sleep loss” from a single transcriptome sample, collected at 7–8 hr or 31–32 hr of time awake. “Within-subject” refers to the classification of “acute sleep loss” from a baseline-corrected transcriptome sample collected at 10–11 hr or 34–35 hr of time awake. Black horizontal line represents the decision boundary for classifying a sample at a probability of 0.5. Blue: samples taken following 1 wk of sufficient sleep; gold: samples taken following 1 week of insufficient sleep, gray: all samples. Data shown based on the final “all features” elastic net model trained on all samples within the “UPUS” training set and applied to all samples within the corresponding independent validation set. ACC = accuracy. (b) Distribution of mRNA abundance values for features selected as classifiers of the sleep debt variable “acute sleep loss” when applying elastic net to “all features” within the “UPUS” training set (without baseline correction). mRNA abundance data is shown for all samples within the “UPUS” training and validation sets with no baseline correction.

The IV classification performance of the identified panels of biomarkers ranged from 75% to 92% (Table 1, Supplementary File S1, Figure 5a). The LOSO-CV performance was similar. The combination of “all features” and elastic net applied to UPUS data produced a panel with the highest classification performance (92% accuracy, MCC = 0.83), due to greater levels of sensitivity (sensitivity = 90% compared with <60% in other UPUS based searches). The performance of the predictors for acute sleep loss appeared similar for the two sleep history conditions (Figure 5a, left panel). For the between participant approaches, panels based on UPUS data tended to be more specific than sensitive, while OPUS-based panels were more sensitive than specific. The number of features identified was not markedly different (68 UPUS, 48 OPUS). Figure 5b demonstrates the robustness of the best performing panel. Within the training set, there is a clear distinction in mRNA abundance levels of all features between samples (participants) of each class. In the corresponding validation set the difference is not so distinct. Some individual features fail to produce the expected signal in the validation set. However, when the entire panel is considered the separation between samples is clear and a performance of 92% accuracy is achieved (Figure 5a). Example mRNA abundance profiles for the biomarker A_23_P205273 (probe targeting a transcript of the gene *PP1R13B*) for “acute sleep loss, between-subject” are shown in Supplementary File S2.

##### Classifying “acute sleep loss” without confounding by circadian phase; within-subject assessment.

To remove the potential confound of circadian phase and possibly improve the classification performance of “acute sleep loss,” we performed a within participant analysis comparing sample #2 (no acute sleep loss) with the 24 hr separated sample #10 (acute sleep loss) after having subtracted sample #1 from both (i.e., correcting for the baseline condition within each participant).

Baseline correction for the variable “acute sleep loss” did not improve upon the performance achieved when using a single, nonbaseline-corrected transcriptome sample (Figure 5*). However, in contrast to the between participant approach, when baseline correction was applied the *a priori* selected features were able to classify samples with similar, or greater accuracy than those selected from “all features.” This is expected as the *a priori* features were selected using within individual analyses.*

#### Biomarker panels for chronic sleep loss

##### Classifying “chronic sleep insufficiency”; between-subject assessment.

For “chronic sleep insufficiency,” we assessed whether it is possible to determine if a sample taken in the afternoon comes from an individual who has had less than 6 hr of sleep over an extended period (1 week). All samples were labeled by the sleep history condition in which they were collected. Training and validation sets comprised sample #1 from each sleep history condition. Where sample #1 was not available for a participant sample #2 was used instead.

**Figure 6.**
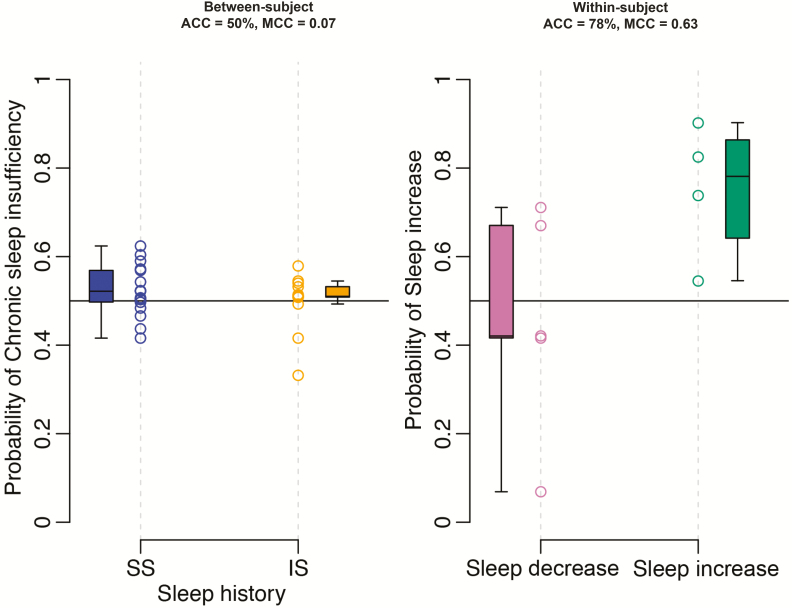
Classification performance for “chronic sleep insufficiency” between- and within-subject. “Between-subject” refers to the classification of a single transcriptome sample to two classes: following sufficient sleep (SS) or following insufficient sleep (IS). Data shown based on the final “all features” elastic net model trained on all samples #1 (or #2) within the “UPUS” training set and applied to all samples #1 (or #2) within the corresponding independent validation set. “Within-subject” refers to the classification of a differential transcriptome sample to two classes: sleep decrease or sleep increase. Data shown based on the final “all features” elastic net model trained on all “visit 1”–“visit 2” samples, using samples #2 (or #3), within the “UPUS” training set and applied to all “visit 1”–“visit 2” samples, using samples #2 (or #3) within the corresponding independent validation set. Black horizontal line represents the decision boundary for classifying a sample at a probability of 0.5. ACC = accuracy.

In contrast to “time awake” and “acute sleep loss,” the better performing panels of biomarkers for “chronic sleep insufficiency” were derived from the “sleep sufficiency” *a priori* selected feature list. However, the highest IV classification accuracy achieved was only 57% (Figure 6a). The structure of the training and validation sets had an impact on performance. For OPUS data, the IV classification performance was worse than random classification (expected accuracy of 50%). This complete misclassification of samples was due to the effect of the individual within the design of the OPUS training and validation sets. The validation samples from a participant providing “sufficient sleep” samples to the OPUS training set were classified as “sufficient sleep” in the IV. Similarly, with “insufficient sleep” samples. Hence, the negative MCC values (range = −0.45 to −0.17). We can only assume this is because elastic net has identified a related, but currently unknown, variable that can stratify participants. For this reason, we see a larger impact on IV performance in the “all features” list, which is reduced when the effect of the individual is removed in LOSO-CV. The number of features selected was similar (9 UPUS, 11 OPUS).

The effects of insufficient sleep on many physiological and behavioral variables are exacerbated by acute sleep deprivation [18]. Therefore, a pertinent question to ask is whether sample #9 (or #10), collected at the same time of day as #1 (or #2), but following a period of total sleep deprivation, emphasizes the changes in the blood transcriptome in response to 1 week of chronic sleep insufficiency and improves classification accuracy. Hence, we repeated the above described analysis with sample #9 (or #10). The panels identified did not improve performance (highest classification accuracy of 57%), even with a greater number of biomarkers within the panel (21 UPUS, 36 OPUS).

##### Classifying “sleep increase/decrease”; within-subject assessment of sufficient versus insufficient sleep.

Clinics/researchers monitoring changes in sleep behavior over time may benefit from having a panel of biomarkers able to identify changes in sleep duration (i.e. an increase or decrease) within an individual. Thus, we investigated whether it is possible to identify individuals who have gained or lost sleep. Within the constraints of our protocol this translates to distinguishing the participants subjected to 1 week of insufficient sleep followed by 1 week of sufficient sleep, from the participants subjected to 1 week of sufficient sleep followed by 1 week of insufficient sleep. We used only the UPUS training and validation sets comprising the difference in mRNA abundance between a sample of the first “visit” and its corresponding sample from the second “visit” within a participant. We searched for biomarker panels for each of the 10 differential sampling points. Where a given sampling was unavailable for a participant the sample from the subsequent sampling point was used, for example, if sample #1 was not available we took sample #2 (for sample #10, sample #9 was used if #10 was not available).

Comparing across sampling points, the highest classification accuracy was provided by sample #2 (or #3), equivalent to sampling between 11:00 and 14:00 hr (Figure 6, Supplementary File S1). Table 1 summarizes results for sample #2 and, as a contrast, sample #9 (or #10). Relatively few features were selected by elastic net from “all features” (36 from #2 [or #3], 62 from #9 or [#10]). Panels based on the “sleep sufficiency” *a priori* features gave the greatest IV classification accuracy (89% and 67% accuracy, 0.8 and 0.49 MCC for samples #1 and #9, respectively). However, the better performance does not appear to be directly related to the increased number of features in the panel (Supplementary Figure S1). The striking difference between LOSO-CV and IV classification performance (ca. 20% difference in accuracy) we expect to be an effect of the reduced number (10 samples in the training set) and variance between samples. Indeed, the high classification performance is not realized as a prominent distinction between classes’ mRNA abundance profiles within the validation set (Supplementary Figure S2). An example of a biomarker for “sleep increase/decrease” is shown in Supplementary File S2.

### Functional annotation of mRNA biomarker panels

We next investigated the process associated with the various panels predicting sleep debt status. All panels of mRNA biomarkers and their associated genes are provided in data set S1. A comparison of the genes associated with each panel is provided in data set S2. Here, we focus on the functional interpretation and overlap of panels identified by (1) *a priori* knowledge, and (2) selected by applying elastic net to UPUS training sets.

#### Biomarker panels for acute sleep loss

##### *a priori* knowledge informed panels for acute sleep loss.

Genes with known function associated with the 26 “hours awake” *a priori* selected features are involved in lipid metabolism (*ABCA1*, *PTPLB* [*HACD2*]), ubiquitination (*HECTD3*, *UBE2V1*), centrosome function and the regulation of mitosis/meiosis (*C6orf204* [*CEP85L*], *C1orf96* [*CCSAP*], *NEK1*, *NCAPD2*), actin binding (*TPM4*), ATPase binding (*TOR1AIP2*), insulin signaling (*STXBP4*), and regulation of cardiomyogenic differentiation [42] and cell proliferation via MAPK, PI3K/AKT, and JAK/STAT signaling (*PARM1*) [43]. Many features were associated with the regulation of transcription/translation; three DNA binding histone proteins (*HIST1H3J*, *HIST1H4J*, *HIST1H4K*), a subunit of the mediator complex that activates RNA polymerase II (*MED9*), a phosphoinositide 3-kinase (*PI3K*) regulatory subunit that regulates mTOR-mediated gene expression and is involved in glucose uptake and insulin signaling (*PIK3R3*), a coiled-coil domain protein that inhibits β-catenin and negatively regulates WNT-driven gene expression (*CCDC88C*), and an RNA methyltransferase that regulates ribosomal biogenesis (*FTSJ3*). The “hours awake” set of features was used as input to four elastic net searches (“time awake” and “acute sleep loss” using within- and between-subject assessments). Ten of the 26 features were selected in three of the four searches, including *PARM1*, *FTSJ3*, *PIK3R3*, *PARM1*, *HIST1H4K*, *C1orf96* (*CCSAP*), and *TOR1AIP2*, with *PARM1* and *FTSJ3* present in all four panels.

##### Panels for acute sleep loss selected from all features.

**Figure 7.**
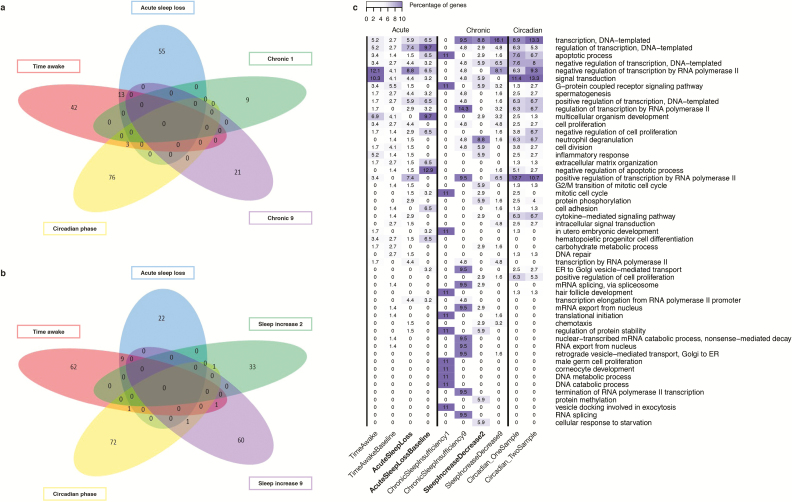
Comparison of biomarker panels for different sleep debt related variables. Comparisons made between panels of biomarkers identified when using “all features” and “UPUS” as training data to elastic net. (a) Comparison by genes associated with biomarkers that can predict/classify between individuals (i.e. one sample based). Chronic 1 refers to biomarkers for “chronic sleep insufficiency” identified when using sample #1 (or #2). Chronic 9 refers to biomarkers for “chronic sleep insufficiency” identified when using sample #9 (or #10). Circadian phase refers to biomarkers for Circadian phase using one sample as defined in Laing et al. [17]. b) Comparison by genes associated with biomarkers that can predict/classify within individuals (i.e. two sample based). Sleep increase 2 refers to biomarkers for “sleep increase/decrease” using sample #2 (or #3), sleep increase 9 refers to biomarkers for “sleep increase/decrease” using sample #9 (or #10). Circadian phase refers to biomarkers for Circadian phase using two samples as defined in Laing et al. [17]. (c) Comparison by associated “top 10 enriched” GO terms, based on the percentage of genes associated with the panel that are associated with a given GO term. Highlighted columns are biomarker panels with >70% classification accuracy when applied to an independent validation set.

There was little overlap of genes associated with features selected as biomarkers for “time awake” and “acute sleep loss” (Figure 7a, between individuals; Figure 7b within individuals). The 58 unique genes associated with biomarkers for predicting “time awake, between-subject” included those related to inflammatory responses (*NFKB2*, *NFKBID*, *CHST2*), T-cell development (*SELK*, *LRRC8A*), ubiquitination (*HECTD3*, *UBE2K*), apoptosis (*PPP1R13B*, *SORT1*, *C14orf153* [*APOPT1*]), RNA splicing/processing (*BRUNOL6*, *NOL8*, *CSDE1*, *POP1*), transcription regulation (*DNMT1*, *REST*, *ZBTB7B*, *TDG*), PI3K signaling (*PIK3R3*, *INPP5F*), mTOR signaling (*DDIT4*), MAPK/PKC/JNK signaling (*DUSP22*), TNF signaling (*TNFRSF12A*), phosphodiesterase activity (*PDE7B*), and muscle actin binding (*PALLD*). Eleven of these genes overlapped with the panel for “time awake, within-subject” (*AF060170*, *AJ009817*, *BRUNOL6*, *CRTAP*, *DUSPSS*, *GAS2L2*, *MPP3*, *POP1*, *PPP1R13B*, *REST*, *UBE2K*).

The 68 genes associated with classifiers of “acute sleep loss, between-subject” included those related to ubiquitination (*HECTD3*, *SPOP*, *ASB6*), apoptosis (*MAK10*, *C14orf153* [*APOPT1*], *SCRIB*), RNA processing (*CSDE1*, *PHAX*, *GIGYF2*, *CDC2L1*), transcription regulation (*TAF2*, *TAF3*, *REST*, *TDG*, *ELL3*, *TCF12*, *ZNF395*, *ZC3H7A*, *GTF3C2*), DNA repair (*PDS5B*, *MCM9*), neuron development/regulation (*NCDN*, *MPP5*, *CDC42*), phosphodiesterase activity (*PDE7B*), muscle actin binding (*PALLD*), TGF/NFKB signaling (*FKBP1A*, *CXXC5*), WNT signaling (*ZNRF3*, *WNT6*), TNF signaling (*TNF*), MAPK/PI3K signaling (*FGR*), and mitochondrial electron transport (*CYCS*). Only three genes were also associated with the panel for “acute sleep loss, within-subject” (*MAK10*, *PPP1R13B*, *REST*).

Thirteen genes were associated with the within-subject panels for “time awake” and “acute sleep loss” (Figure 7a) and included *CRTAP*, *TDG*, *PPP1R13B*, *C14orf153*, *HECTD3*, *PALLD*, *MPP3*, *PDE7B*, *CSDE1*, and *REST*. Although little genetic overlap, there was greater similarity in the GO terms associated with all panels for “time awake” and “acute sleep loss” (Figure 7c). Top ranked GO terms common to the lists include regulation of transcription, apoptotic process, negative regulation of transcription from RNA polymerase II, and signal transduction.

#### Biomarker panels for chronic sleep loss

##### *a priori* knowledge informed panels for chronic sleep loss.

Five hundred and six features were selected *a priori* as biomarkers for predicting/classifying sleep sufficiency. An interaction network analysis of the 506 features showed that there were interconnected nodes related to transcription/translation, RNA processing/transport, chromatin modification/transcription mediation, DNA replication/repair, protein ubiquitination and degradation, and mitochondrial metabolic pathways (Supplementary Figure S3). The node with the largest number of interactions (edges) in the network is *RIPK4*, a serine/threonine kinase that interacts with protein kinase C-delta and also activates *NFKB*. Few of these *a priori* features were selected by elastic net to classify “chronic sleep insufficiency” and “sleep increase/decrease,” with little overlap between the four panels identified. Three genes were associated with two of the four lists; *STRADB* (component of complex that activates serine/threonine kinase 11 and regulates apoptosis via JNK1 signaling), *NDUFC1* (subunit of mitochondrial NADH dehydrogenase complex 1), and *SIAH2* (E3 ligase involved in protein ubiquitination/degradation).

##### Panels for chronic sleep loss selected from all features.

Given all features, elastic net selected nine with which to classify “chronic sleep insufficiency” based on sample #1 (or #2). Associated genes with known function were related to ubiquitination (*SUGT1*), exocytosis (*EXOC6B*), translation initiation (*EIF2S2*), DNA catabolism (*DNASE1L2*), heat shock protein response (*DNAJC9*), and Rho signaling (*ARHGEF6*). The 21 features selected to classify “chronic sleep insufficiency” based on sample #9 (or #10) were related to genes involved in RNA splicing/processing (*CDC40*, *DCP1B*, *NCBP2*), transcription regulation (*GABPB1*, *BRMS1L*), muscle actin binding (*TPM3*), cell division (*POGZ*), Golgi protein transport (*BLZF1*, *ARFGAP1*), acetyltransferase activity (*LPCAT1*), ubiquitination (*SUMO2*, *SUGT1L1*), and proteasome assembly (*POMP*). There was no overlap between the two panels for “chronic sleep insufficiency” (Figure 7a).

Thirty-four features were selected for the classification of “sleep increase/decrease” using sample #2 (or #3). These were related to genes involved in immune/inflammatory response (*C4B*, *CCL25*), apoptosis (*ELMO3*), ubiquitination (*UBE2F*, *UBQLN1*, *KLHL24*), lysosomal protein degradation (*LAMP2*), transcription regulation (*ZNF397*), Golgi protein transport (*STK25*, *ERGIC3*), translation regulation (*EEF1A1*), endoplasmic reticulum organization (*KIAA1715*), WNT signaling (*PFTK1*), cell proliferation (*ODC1*), mitosis regulation (*NUSAP1*, *ENSA*), heat shock protein response (*HSPA8*), and G protein signaling (*GPR27*). This set of genes did not overlap with genes associated with the 62 features selected for classifying “sleep increase/decrease” using sample #9 (or #10) (Figure 7b). However, at the level of biological function, the panel for “sleep increase/decrease” using sample #9 (or #10) overlapped with other panels (e.g. transcription regulation [*ZNF395*, *ZSCAN20*, *ZNF620*, *WHSC1*, *RUNX3*, *PPHLN1*, *BATF2*], muscle actin binding [*TPM4*], neuron development [*SERPINF1*, *S1PR5*, *NRG1*], NFKB signaling [*MTDH*], JNK signaling [*MAGI3*], heat shock response [*HSF2*], and G protein signaling [*RGS9*]), in addition to unique functions (e.g., calcium transport [*TMEM38B*, *LETM1*, *CACNB4*, *CACNA2D2*]). One gene, *RALGPS1*, which regulates Ral protein signaling, was associated with panels for classifying “sleep increase/decrease” using sample #9 (or #10) and predicting “time awake, within-subject” (Figure 7b).

### Similarities between biomarker panels for acute and chronic sleep loss

There was some overlap in the top ranking GO terms associated with the biomarker panels for chronic sleep loss and those for acute sleep loss (e.g. transcription, DNA template, apoptotic process, signal transduction), but those associated with chronic sleep loss panels were also enriched for terms not found or not highly represented in the acute sleep loss panels (e.g. mitotic cell cycle, neutrophil degranulation, mRNA splicing, termination of RNA polymerase II transcription, mRNA export from nucleus, translation initiation, regulation of protein stability) (Figure 7c). To make the distinction between GO processes associated with good and poor performing biomarker panels, we have highlighted the good performing panels in Figure 7c.

An interaction network of the genes associated with the combined panels for acute and chronic sleep loss shows that, apart from one gene (*ZNF395*), there is no overlap, although they are connected around some common and also unique biological functions (Supplementary Figure S4). Circadian clock genes are associated with acute and chronic sleep loss and are connected by one of the most connected nodes in the network, BTRC, which is the F-box ubiquitin ligase that mediates the degradation of PERIOD (PER) protein. Ubiquitination is common to many of the acute sleep loss related panels (e.g. *HECTD3*, *UBE2K*), and also appears in panels related to chronic sleep loss, albeit based on different genes (e.g. *UBE2F*, *SUMO2*, *SUGT1*, *UBQLN1*). The regulation of transcription is also common to both, but RNA processing and translation is more restricted to chronic sleep loss (e.g. *EEF1A1*, *EIF4B*, *EIF2S2*). Heat shock response proteins (*HSPA8*, the most connected node, *HSF2*, and *DNAJC9*), calcium transport (*CACNA2D2*, *CACNB4*) and tropomyosin muscle actin binding (*TPM3*, *TPM4*) are also only found associated with chronic sleep loss. Likewise, TNF signaling (*TNF*, *TNFRSF12A*) and PI3K signaling (*PIK3R3*) are restricted to acute sleep loss.

### Lack of overlap between circadian markers and sleep debt markers

We next investigated whether the mRNA biomarker panels for sleep debt status were distinct from the previously identified mRNA biomarkers for circadian phase [17, 44]. Across all approaches (*a priori*, elastic net applied to UPUS and OPUS training sets), we identified 1,195 unique genes/transcripts related to biomarkers for sleep-debt related variables. Only 23 of these (i.e. ~2%) overlap with genes previously reported as relevant for assessing circadian phase (data set S3) [17, 44]. Even if we exclude the *a priori* selected genes from this comparison because by definition they exclude circadian genes, the overlap is only 21 out of 702 genes (data set S3). It is also worth noting that approximately one-third of the biomarker transcripts for circadian phase did not have circadian expression profiles (as defined in refs. [18, 21]). The lack of overlap between the circadian phase biomarkers and those selected from all features for acute and chronic sleep loss is shown in Figure 7, a and b. This indicates that transcriptional processes activated by sleep debt (or sleep homeostasis) are very different from those under circadian control. Of the 1,195 only 156 were identified in more than one independent search (comparing *a priori* panels with those identified from “all features” by elastic net, data set S3). Only 3 of the 156 were also related to circadian phase (*C17orf55*, *DDIT4*, *LDLR*). Indeed, transcriptome biomarkers previously identified to predict circadian phase are more related to blood cell function (immune function) and glucocorticoid signaling (inflammatory responses) [17] than to the functional processes associated with the sleep loss biomarkers identified here, which are more generally related to cellular stress responses.

## Discussion

The results show that biomarker panels derived from the blood transcriptome can, on the basis of one blood sample, reliably identify whether an individual has skipped one night of sleep, even when no baseline data of that participant are available. The data also show that when using blood transcriptome derived biomarkers, assessment of chronic sleep insufficiency, defined as less than 6 hr, is more challenging. However, it may be possible to assess changes in sleep sufficiency within an individual. Comparison of various validation and feature selection approaches and several measures of performance supports the robustness and validity of the identified biomarker panels. Functional annotation of the identified mRNA biomarkers indicates that activation of the cellular stress response is central to the consequences of sleep loss.

## Performance of mRNA biomarker panels for various categories of sleep debt

### Acute sleep loss

Performance of mRNA biomarkers for classification of time awake in broad categories, that is, wakefulness of more or less than 24 hr, or prediction of sleep loss or not, reached respectable levels (e.g. 92% for the between-subject assessment of “acute sleep loss”). This contrasts with the poor performance of mRNA biomarkers for predicting how many hours an individual has been awake (i.e. within a range of 7–34 hr), even when a within-subject comparison was applied. The latter observation may imply that the blood transcriptome response to time awake is too weak or variable between subjects, or too much confounded by circadian variation to make a parametric prediction of hours awake [19]. The latter interpretation is supported by a nonlinear time course of the predictor with time awake (Figure 3). In the prediction of broader categories this circadian confound is reduced (“wakefulness of >24 hr”) or completely removed (“acute sleep loss”). This may indeed explain the better performance in the classification of broader categories of acute sleep loss. Further improvement of parametric prediction may require a combination of biomarkers for assessment of circadian phase, which we have shown to be feasible [17], with the current panels for “time awake.” Alternatively, other machine learning approaches that consider nonlinear relationships between features and variables may be more appropriate. Overall, the results demonstrate that the blood transcriptome contains information to assess acute sleep loss status even in a between-subject assessment, that is, without prior information on the blood transcriptome of an individual. Importantly, performance of these predictors was not affected by the prior sleep condition. In other words, the performance of the biomarker for acute sleep loss was good regardless of whether the total sleep deprivation was preceded by a week of sufficient or insufficient sleep (Figure 5a, left panel).

### Chronic sleep insufficiency

Chronic insufficient sleep in an individual could only be reliably identified on the basis of the blood transcriptome when the “sufficient sleep” blood transcriptome information for that individual was available. This limitation most likely reflects the rather small effects of 1 week of insufficient sleep and the rather large between-subject variation in the blood transcriptome [18]. Less than 6 hr of sleep should be considered insufficient sleep as many epidemiological studies have used 6 hr as a cut off below which adverse health outcomes are reported. Furthermore, robust changes in sleepiness and neurobehavioral performance have been observed under these conditions [2]. It may be argued that 1 week does not represent a chronic condition as far as adverse long-term health outcomes are concerned and that longer interventions are needed to identify relevant mRNA biomarkers. Furthermore, in the present analysis, we contrast insufficient (~5.7 hr assessed by PSG) and sufficient (~ 8.5 hr assessed by PSG) sleep which was achieved by a 6 and 10 hr sleep opportunity, respectively. Thus, it may be argued that a realistic baseline condition (8 hr sleep opportunity) would have provided additional information. Nevertheless, the current approaches have identified biomarkers that can be used to assess changes in chronic sleep debt status.

## Genes associated with panels of biomarkers

Because the identified biomarkers for sleep loss are derived from the whole blood-based transcriptome that includes mRNA from all blood cell types, a naïve expectation might be that transcripts would necessarily be related to blood cell functions such as immune responses. However, blood interacts with all tissues and organs and represents a window for assessing whole-body function. Thus, gene expression in whole-blood can include and respond to a broad range of biological functions, as we have previously shown [18, 21]. In fact, few of the biomarkers that we have identified here are directly associated with immune function, although there are recurrent links with NFKB inflammatory signaling in the various biomarker panels. Indeed, whole blood appears to respond to sleep deprivation in ways similar to those observed in other tissues. Sleep deprivation and extended wakefulness increase energy demands and accumulated oxidative stress. Many sleep deprivation studies have found an overall down-regulation of many genes, presumably as a cellular response to limit energy expenditure and reduce associated levels of cellular stress [45]. Sleep deprivation has consistently been found to induce cellular stress responses and the unfolded protein response (UPR) and subsequent endoplasmic reticulum-associated protein degradation (ERAD) in response to energy deprivation and oxidative stress [46]. This leads to upregulation of gene transcription and protein translation, increased heat shock protein chaperone activity in response to misfolded proteins, higher levels of protein ubiquitination, trafficking and degradation, increased cytokine-mediated inflammatory responses, and ultimately cell injury and apoptosis (e.g. Naidoo [47]). Many of the biomarkers that we have identified for sleep loss in this study are directly related to these cellular responses, including markers for specific mitochondrial metabolic pathways.

Short sleep duration in adults has previously been associated with biomarkers for inflammation [48, 49], including TNF which was identified as a biomarker for acute sleep loss in the current study. The misfolded protein response heat shock protein HSP70 is upregulated in the rat brain following sleep deprivation [50] and also in patients with sleep apnea [51]. HSPA8 is a member of the HSP70 family of proteins and was identified as a biomarker for chronic sleep loss in this study. DNA damage induced by cellular stress is also increased after partial and total sleep loss in rats [52] and genes for several DNA repair proteins were found in biomarker panels for both acute and chronic sleep loss. Mitochondrial enzymes have been shown to be biomarkers in rats for sleep deprivation in response to higher cellular energy demands required during prolonged wakefulness [53]. In the present study, we identified three NADH ubiquinone oxidoreductase subunits (*NDUFC1*, *NDUFS1*, *NDUF89*) and a succinate dehydrogenase subunit (*SDHD*) as biomarkers for chronic sleep loss, and cytochrome C (*CYCS*) as a biomarker for acute sleep loss. There is also a growing literature showing that sleep deprivation impairs adult neurogenesis (e.g. hippocampal-associated learning [54]) and several biomarkers for sleep loss were associated with neuronal development and regulation in this study.

Amylase has previously been identified as a highly correlated biomarker for sleep drive in drosophila, and saliva enzyme activity and RNA abundance also correlated with sleep deprivation in humans [13]. Mannosidase is structurally very similar to amylase and both degrade polysaccharides [55]. The list of 506 *a priori* features for “sleep sufficiency” was enriched for the GO molecular function “mannosidase activity” (MAN2A1, MAN2A2, MAN2B2, KIAA2018). Amylase gene expression in humans is specific to saliva and the pancreas and it could be that mannosidase activity in human blood represents an equivalent blood-based biomarker for chronic sleep loss. Diacylglycerol (DAG 36:3) has also been identified as a marker for sleep debt in both rats and humans [14]. It is worth noting that while we did not identify any DAG-related transcripts (e.g. DAG kinase or lipase) in our biomarker panels, it is known that DAG can be converted to phosphatidic acid and thus regulate mTOR signaling via PI3K pathways [56, 57], both of which we see represented in our sleep loss biomarker panels.

Adenosine is a known mediator of sleep-promoting effects of wake and a potential biomarker for prolonged wakefulness [58], but we do not see any adenosine-related transcripts in our panels for acute or chronic sleep loss. We do however identify the GABA type A receptor (*GARBRP*) and a GABA type A receptor associated protein (*GABARAPL2*) as potential biomarkers for chronic sleep loss. GABAergic neurons in the brain regulate sleep [59] and numbers of GABA receptors in those neurons change in response to sleep deprivation [60].

Finally, it should be noted that many of the genes associated with identified biomarkers have a role in muscle and cardiac function, with links to cardiovascular disease (e.g. *SELK* protects cardiomyocytes from stress, *INPPSF* modulates cardiac response to stress, *TAF3* regulates myocyte differentiation, *THBS4* regulates myocardial function, *ANK3* regulates cardiac muscle contraction, and *MAK10* regulates smooth muscle proliferation). Short sleep has been shown to be associated with cardiovascular disease [61]. Also, because many transcripts have roles in transcription/translation and cell proliferation/apoptosis there are links with a range of different cancers, which may be related to sleep loss and increased risk of cancer observed in shift workers [3]. *REST* also appears in several of the acute sleep loss lists and, in addition to being a suppressor of neuronal genes in non-neuronal cells, protects neurons from oxidative stress and amyloid β-protein toxicity [62]. *REST* is down-regulated in Alzheimer’s and its expression levels correlate with longevity [62]. Thus, in addition to representing robust and reliable biomarkers for sleep status, the identified RNA abundance features may also be associated with the long-term adverse effects of sleep disruption.

## Specificity of identified blood mRNA biomarkers of sleep debt and practicality of use.

Sleep, circadian processes and external (i.e. lifestyle and environmental) factors all interact. In our protocol, the effects of external factors were minimized as we assessed a cohort of healthy individuals of similar age, under clinically controlled conditions. However, samples collected across the constant routine describe changes in the transcriptome in response to sleep debt that also include circadian modulation. We thus considered the timing of samples, focused on those comparisons that removed the confounding effects of circadian processes, to identify specific biomarkers of sleep debt.

The usefulness of these biomarkers in the real world will depend on various factors. A concern is that these biomarkers are not just affected by sleep debt parameters but also health status which is likely to affect immune function. However, when comparing all panels, it is encouraging to note that not many of the identified biomarkers are associated with immune responses. In fact, the majority of the biomarkers are associated with more generalized cellular functions, and in particular, the regulation of transcription and translation, including common signaling pathways such as *WNT*, *mTOR*, and *PI3K*. Other common themes included protein transport/degradation and ubiquitination, apoptosis, mitosis and cell proliferation, neuronal development and function, and aspects of skeletal and cardiac muscle function. Hence, as the panel of biomarkers was associated with multiple processes, we expect their predictions/classifications to be more robust than the single molecules reported to date [13, 14]. Similarly, other biomarkers which associate with lipid metabolism, glucose transport, and insulin responses may also be sensitive to individual differences in feeding and fasting and we did identify features that are associated with these responses. In particular, elements of the *PI3K* signaling pathway that also regulates insulin signaling were common to many of the panels for the classification of “acute sleep loss.” Determining a final panel of biomarkers (and associated prediction/classification model) for real world application will therefore require further assessment of the population, including different conditions to those assessed in our protocol, and the acceptable trade-off between sensitivity and specificity to be defined. The blood transcriptome and the response to sleep loss varies with age [63]. Therefore, it will also be important to investigate the performance of these mRNA biomarkers in other age groups.

## Methodological considerations

*We used a multitude of approaches related to feature selection, creation of training and validation sets, validation methods and used various indices of biomarker performance, with the aim to create transparency about the robustness of our results and their dependency on the applied methods.*

### Use of *a priori* knowledge versus prefiltering versus unbiased approaches.

Identifying panels of biomarkers for “time awake” and “acute sleep loss” between individuals (no baseline correction), it was clear that there is an advantage in using “all features” as input (6%–15% higher performance for UPUS data). However, this advantage was reduced when considering the individual (“acute sleep loss, within-subject”). This we expect to be due to our approach to selecting the *a priori* selected features, which rests on the within participant analysis we conducted and reported in Möller-Levet et al. [18]. Conversely, for variables related to sleep sufficiency, we found prior knowledge to be beneficial. While this is likely due to the selection criteria applied, in that the entire time series and data set were considered in our *a priori* selection, our work highlights the need to assess different sets of input features, with and without filtering, for biomarker discovery.

### Creating training and validation sets: UPUS versus OPUS.

The available data include many samples (496) but few unique individuals (36). Such data pose a challenge for machine learning. Thus, we used the “gold standard” approach to create independent training and validation sets that comprise samples from distinct participants (UPUS). The disadvantage to this approach is that the number of participants to “learn” from is reduced and may not be representative of the population. We therefore investigated an alternative approach (OPUS) that increased the number of individuals within a training set. Due to the differences in data set structures (differing number of features, participants) Elastic net applied to each training set identified different panels of biomarkers, with varying degrees of overlap (data set S2). We would expect these differences to be reconciled with the availability of a greater number of samples/participants. Nevertheless, to further understanding of the molecular mechanisms associated with sleep debt the biomarkers identified by different approaches will be of interest.

### Validation methods.

The entire data set consists of many highly correlated samples, where samples from the same individual look most similar to each other due to the “trait-like” characteristics of the human blood transcriptome [17, 27]. If not handled correctly this similarity can lead to optimistic measures of performance, even though our algorithms are “blind” to the participants from which transcriptomes originated. Thus, we used different approaches to validate performance. Comparing LOSO-CV and IV performance of each search revealed, in general, similar values. This implies that the procedures implemented adequately prevented overfitting and controlled for optimism in our assessment of performance. Several features of our workflow will have aided this; two different cross-validation strategies were employed, meaning that our “tuning” of hyper-parameters and assessment of performance were independent [25], the 10-fold cross-validation was repeated a large number of times [26], and we considered the “trait-like” behavior of -ome samples [27] and performed LOSO-CV. Our use of different metrics to assess classification performance was necessary as each approach and hence validation set was different. Our use of the unbiased (to size of classes) metric of MCC enabled the comparison of each panel despite differences in class sizes. Reporting sensitivity and specificity is relevant to the selection of a panel for future real-world applications.

## Weaknesses and strengths of the current approach

The results are based on carefully controlled laboratory studies in healthy young volunteers. This is a weakness because it obviously leaves open the question of generalizability to the broader population in real life situations. It is also a strength because the to-be-predicted sleep debt parameters were carefully quantified. Sleep debt parameters were manipulated within a physiological range. It can be argued that more extreme manipulations, for example, 72 hr awake or sleep restriction to 4 hr or less, would have identified more specific biomarkers. On the other hand, biomarkers identified in more extreme protocols may not be relevant to most real-world situations, in which very few people will be awake for 72 hr or sleep less than 4 hr for a week. Another weakness relates to the small sample size. The results from the IV indicate the identified biomarkers are able to generalize to independent samples and are therefore unlikely to reflect a response unique to a particular group of participants. However, without further validation, we are unable to assess the robustness and inter- and intraindividual variability of the selected markers. A strength of the current analyses is that we have quantified the ability of a panel of biomarkers derived from one (or two) samples to predict an individual’s sleep debt status rather than simply listing features that on average change with sleep debt parameters.

## Concluding remarks

Here, we present the first multivariate search and validation of panels of human blood transcriptome biomarkers for sleep debt related variables. Though unable to identify a panel of biomarkers capable of accurately predicting “time awake” (maximum *R*^2^ across methods of 0.29), we did identify biomarkers able to classify an individual as having “acute sleep loss” with 92% accuracy. Accurately discriminating between individuals who have experienced insufficient sleep (5.75 hr) from those who have had sufficient sleep (8.5 hr) we consider to still be a challenge (maximum accuracy achieved here of 57%). However, within an individual, it is possible to identify an increase/decrease in sleep sufficiency with 89% accuracy, even though the average difference in sleep duration was relatively small (2.8 hr). Many of the biomarkers for sleep loss that we have identified are related to cellular responses to stress known to be induced by sleep deprivation and include previously identified single feature biomarkers.

## Supplementary Material

zsy186_suppl_Supplementary_Figure_S1

zsy186_suppl_Supplementary_Figure_S2

zsy186_suppl_Supplementary_Figure_S3

zsy186_suppl_Supplementary_Figure_S4

zsy186_suppl_Supplementary_Table_S1

zsy186_suppl_Supplementary_File_S1

zsy186_suppl_Supplementary_File_S2

zsy186_suppl_Supplementary_DataSet_S1

zsy186_suppl_Supplementary_DataSet_S2

zsy186_suppl_Supplementary_DataSet_S3

zsy186_suppl_Supplementary_Legends
