# Erratum to: Identifying and validating blood mRNA biomarkers for acute and chronic insufficient sleep in humans: a machine learning approach

**DOI:** 10.1093/sleep/zsz313

**Published:** 2020-01-17

**Authors:** Emma E Laing, Carla S Möller-Levet, Derk-Jan Dijkand, Simon N Archer

In this article [Sleep, Volume 42, Issue 1, January 2019, zsy186, https://doi.org/10.1093/sleep/zsy186], Supplementary Figures S3 and S4 were not available on initial publication. Both figures have now been included as Supplementary data.

