# Supplementary figures and images for: Identifying and validating blood mRNA biomarkers for acute and chronic insufficient sleep in humans: a machine learning approach
Source: Sleep. 2018 Sep 24;42(1):zsy186. doi: 10.1093/sleep/zsy186 (PMC6335875; doi:10.1093/sleep/zsy186)

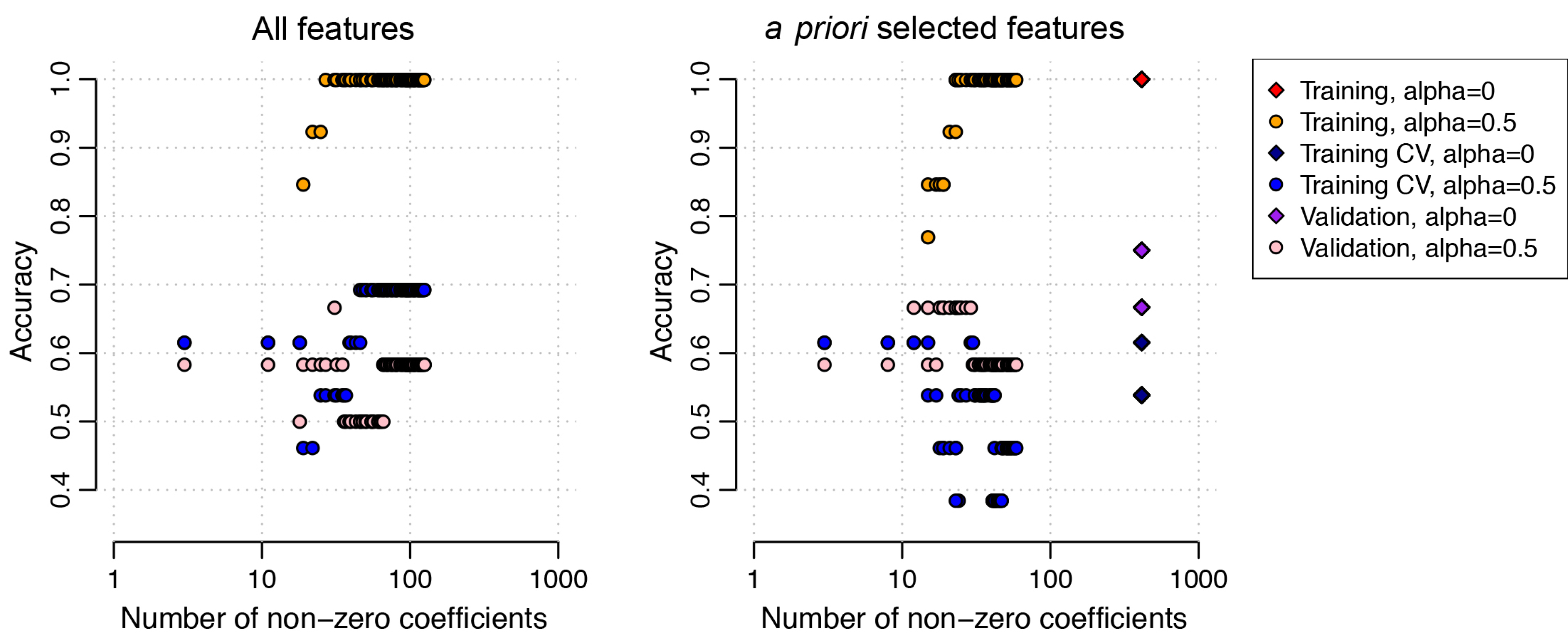

Supplement: zsy186_suppl_Supplementary_Figure_S1 [file zsy186_suppl_supplementary_figure_s1.jpeg]

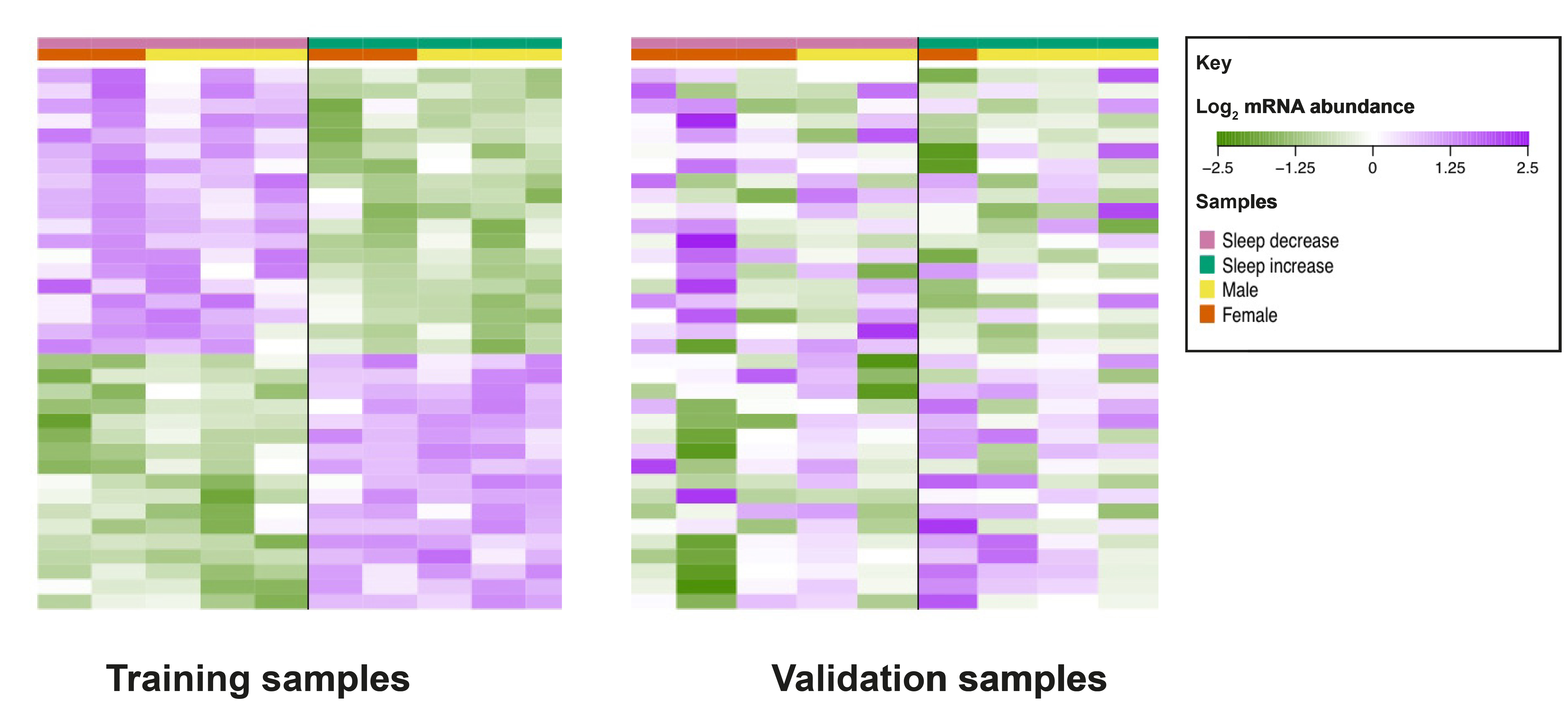

Supplement: zsy186_suppl_Supplementary_Figure_S2 [file zsy186_suppl_supplementary_figure_s2.jpeg]

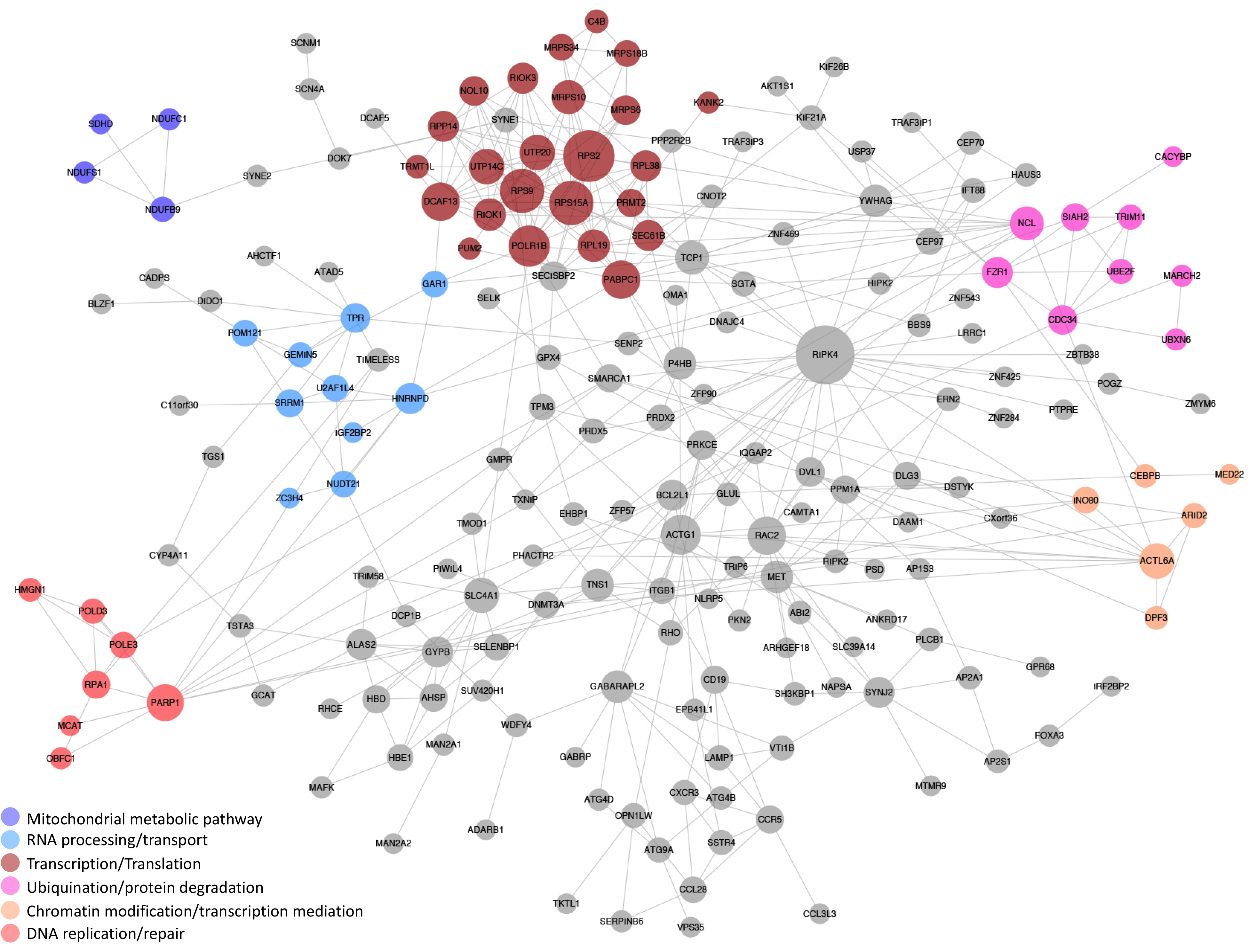

Supplement: zsy186_suppl_Supplementary_Figure_S3 [file zsy186_suppl_supplementary_figure_s3.jpeg]

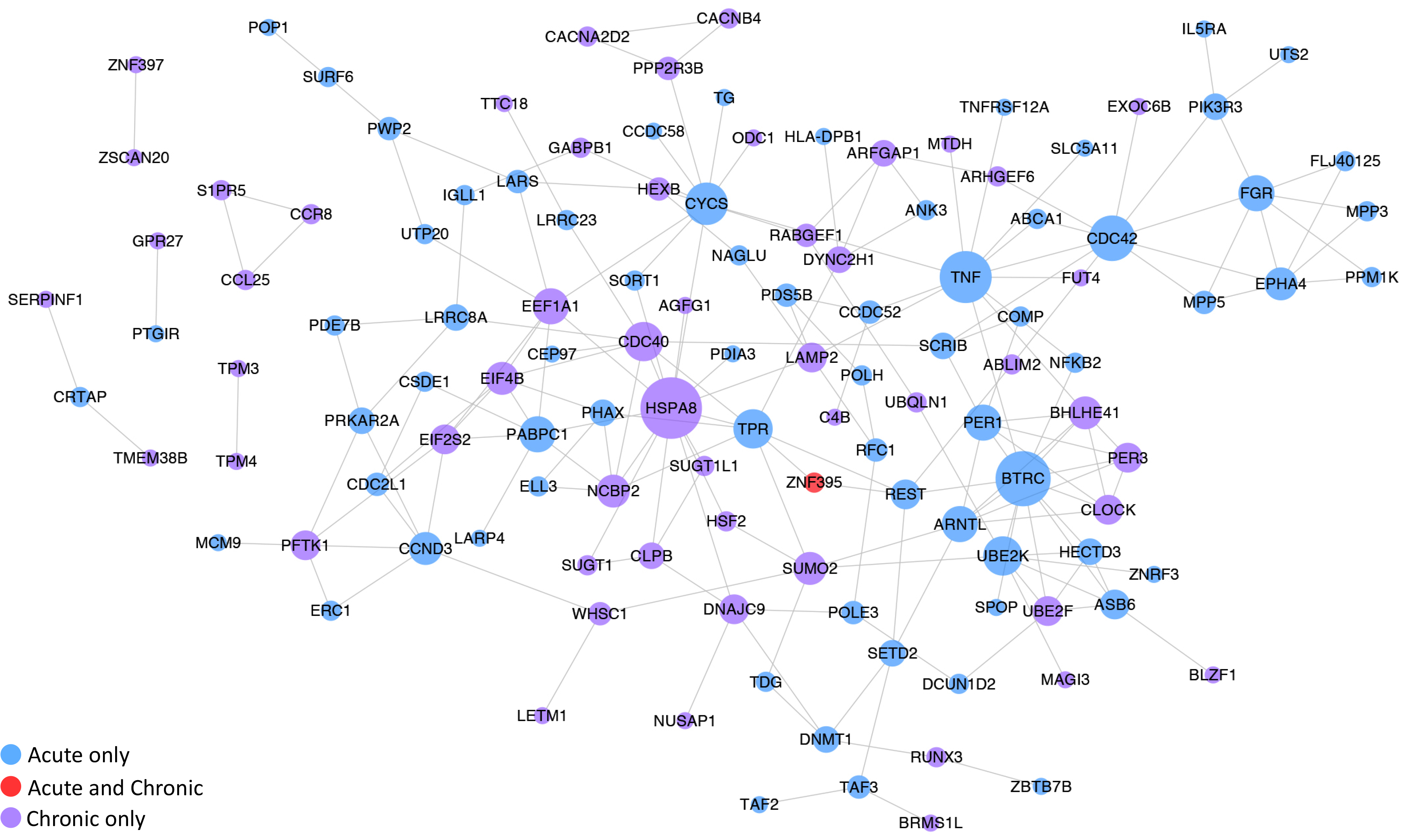

Supplement: zsy186_suppl_Supplementary_Figure_S4 [file zsy186_suppl_supplementary_figure_s4.jpeg]
