# Supplementary material for: Identifying and validating blood mRNA biomarkers for acute and chronic insufficient sleep in humans: a machine learning approach
Source: Sleep. 2018 Sep 24;42(1):zsy186. doi: 10.1093/sleep/zsy186 (PMC6335875; doi:10.1093/sleep/zsy186)
Supplement: zsy186_suppl_Supplementary_Table_S1 [file zsy186_suppl_supplementary_table_s1.docx]

| Biomarkers for | | Number of samples within the training and independent validation sets | Number of features (unique genes) in the final model | | | Leave-One-Subject-Out Cross-validated (LOSO-CV) performance | | | Independently validated (IV) performance | | |
| --- | --- | --- | --- | --- | --- | --- | --- | --- | --- | --- | --- |
|  |  |  | *a priori* knowledge and Ridge | *a priori* knowledge and  Elastic-net | All features and  Elastic-net | *a priori* knowledge and Ridge | *a priori* knowledge and  Elastic-net | All features and  Elastic-net | *a priori* knowledge and Ridge | *a priori* knowledge and  Elastic-net | All features and  Elastic-net |
| Acute sleep loss variables | Prediction of ‘Time awake, between-subject’; samples #1 to #10 | Training = 238 Validation = 234 | 26 (26) | 16 (16) | 211 (205) | R^2^ = 0.29 | R^2^ = 0.34 | R^2^ = 0.45 | R^2^ = 0.28 | R^2^ = 0.29 | R^2^ = 0.39 |
|  | Prediction of ‘Time awake, within-subject’; sample #2- sample #1, #3 - #1, #4 - #1, #5 - #1, #6 - #1, #7 - #1, #8 - #1, #9 - #1, #10 - #1 | Training = 197 Validation = 177 | 26 (26) | 18 (18) | 184 (180) | R^2^ = 0.31 | R^2^ = 0.26 | R^2^ = 0.36 | R^2^ = 0.15 | R^2^ = 0.15 | R^2^ = 0.27 |
|  | Classification of ‘Wakefulness of more than 24 h, between-subject’; all samples, based on the predicted ‘Time awake’ value | Training = 238 Validation = 234 | 26 (26) | 16 (16) | 211 (205) | ACC = 71%  Sn = 56% Sp = 82% MCC = 0.39 | ACC = 74%  Sn = 60% Sp = 84% MCC = 0.46 | ACC = 77%  Sn = 68% Sp = 84% MCC = 0.52 | ACC = 75%  Sn = 63% Sp = 84% MCC = 0.48 | ACC = 76%  Sn = 63% Sp = 85% MCC = 0.49 | ACC = 79%  Sn = 69% Sp = 87% MCC = 0.57 |
|  | Classification of ‘Acute sleep loss, between-subject’; samples #1 vs sample #9 | Training = 49 Validation = 50 | 25 (25) | 15 (15) | 48 (48) | ACC = 82%  Sn = 71% Sp = 89% MCC = 0.62 | ACC = 84%  Sn = 76% Sp = 89% MCC = 0.67 | ACC = 90%  Sn = 86% Sp = 93% MCC = 0.79 | ACC = 82%  Sn = 86%  Sp = 79% MCC = 0.64 | ACC = 78%  Sn = 81%  Sp = 76% MCC = 0.56 | ACC = 88%  Sn = 95%  Sp = 83% MCC= 0.77 |
|  | Classification of ‘Acute sleep loss, within-subject’; difference between sample #2 and #1 vs difference between sample #10 and #1 | Training = 45 Validation = 39 | 25 (25) | 11 (11) | 40 (40) | ACC = 91%  Sn = 96% Sp = 84% MCC = 0.82 | ACC = 91%  Sn = 96% Sp = 84% MCC = 0.82 | ACC = 91%  Sn = 92% Sp = 89% MCC = 0.82 | ACC = 82%  Sn = 68%  Sp = 100% MCC = 0.69 | ACC = 79%  Sn = 64%  Sp = 100% MCC= 0.66 | ACC = 82%  Sn = 68%  Sp = 100% MCC = 0.69 |
| Chronic sleep loss variables | Classification of chronic sleep insufficiency; samples #1 or #2 for insufficient sleep vs samples #1 or #2 for sufficient sleep | Training = 30 Validation = 30 | 408 (408) | 17 (17) | 11 (11) | ACC = 43%  Sn = 0% Sp = 80% MCC = -0.32 | ACC = 43%  Sn = 23% Sp = 60% MCC = -0.18 | ACC = 93%  Sn = 92% Sp = 93% MCC = 0.86 | ACC = 50%  Sn = 0% Sp = 94% MCC = -0.17 | ACC = 41%  Sn = 27% Sp = 53% MCC = -0.21 | ACC = 28%  Sn = 20% Sp = 35% MCC = -0.45 |
|  | Classification of chronic sleep insufficiency; samples #9 or #10 for insufficient sleep vs samples #9 or #10 for sufficient sleep | Training = 33 Validation = 31 | 420 (420) | 36 (36) | 36 (36) | ACC = 42%  Sn = 19% Sp = 65% MCC = -0.19 | ­ACC = 48%  Sn = 50% Sp = 47% MCC = -0.03 | ACC = 64%  Sn = 56% Sp = 71% MCC = 0.27 | ACC = 39%  Sn = 29% Sp = 50% MCC = -0.21 | ACC = 48%  Sn = 47% Sp = 50% MCC = -0.03 | ACC = 23%  Sn = 24% Sp = 21% MCC = -0.55 |
