# Supplementary material for: Identifying and validating blood mRNA biomarkers for acute and chronic insufficient sleep in humans: a machine learning approach
Source: Sleep. 2018 Sep 24;42(1):zsy186. doi: 10.1093/sleep/zsy186 (PMC6335875; doi:10.1093/sleep/zsy186)

Pages 2-7 provide the prediction/classification results for all approaches taken. Layout of the figures :
Upper panel; *a priori* + Ridge, *a priori* + Elastic-net, All features + Elastic-net trained on the Unique participants and samples training set and applied to the corresponding validation set.
Lower panel; a priori + Ridge, a priori + Elastic-net, All features + Elastic-net trained on the Overlapping participants and unique samples training set and applied to the corresponding validation set.

Pages 8-17 provide the classification results for all time points when classifying sleep increase/decrease. Layout of the figures; *a priori* + Ridge, *a priori* + Elastic-net, All features + Elastic-net trained on the Unique participants and samples training set and applied to the corresponding validation set.

Time awake, between-subject

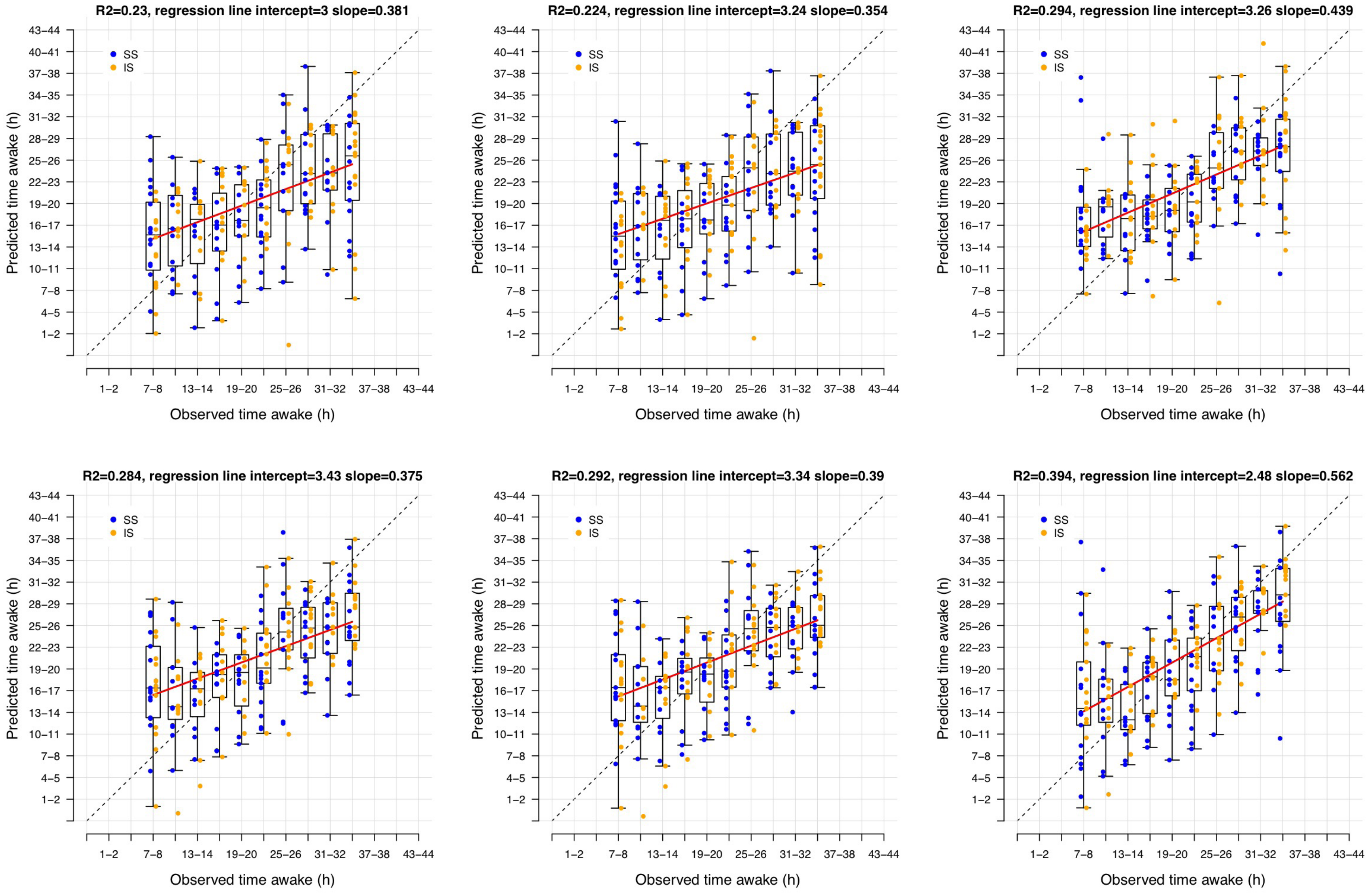

Time awake, within-subject

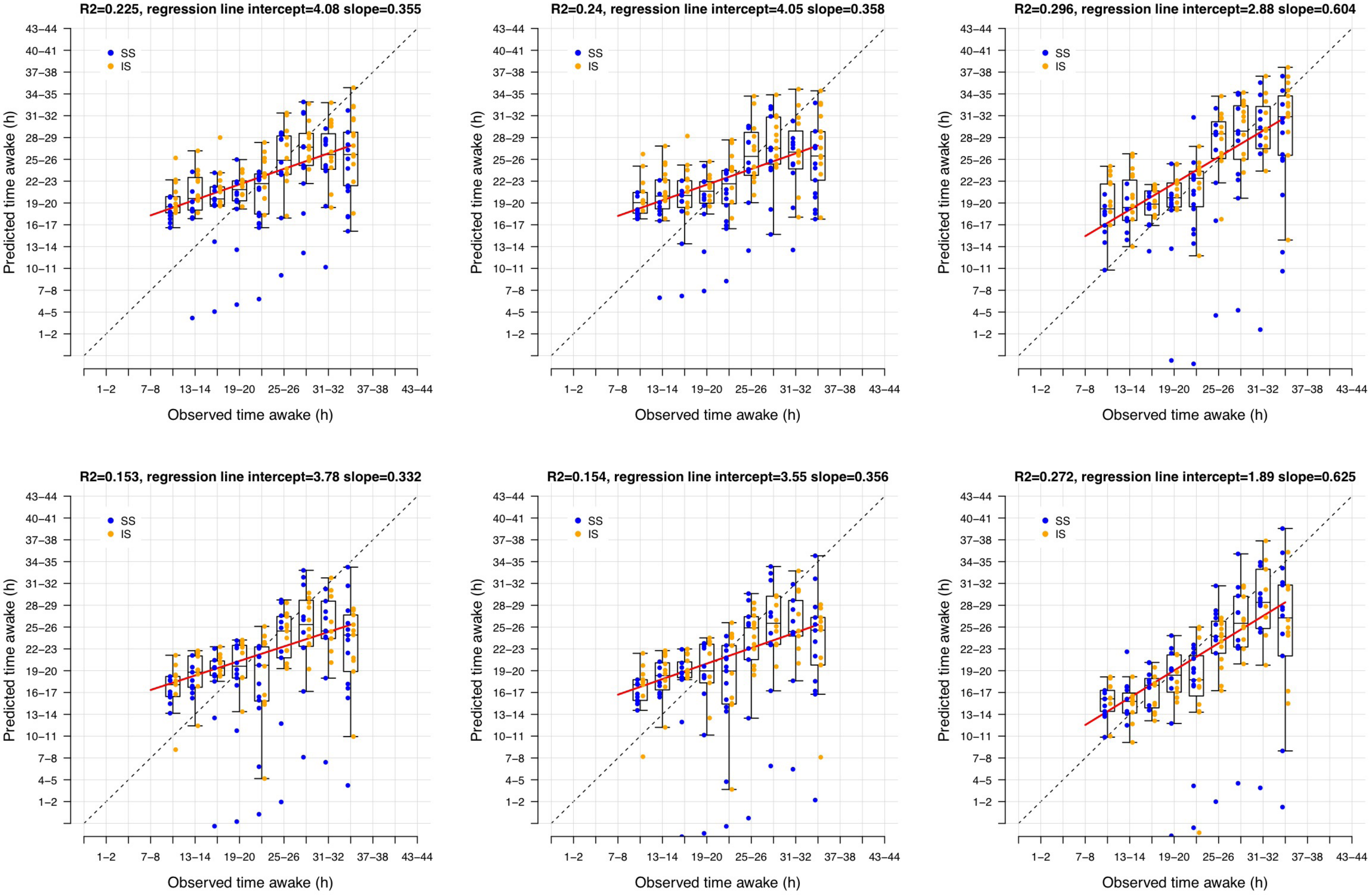

Acute sleep loss, between subject

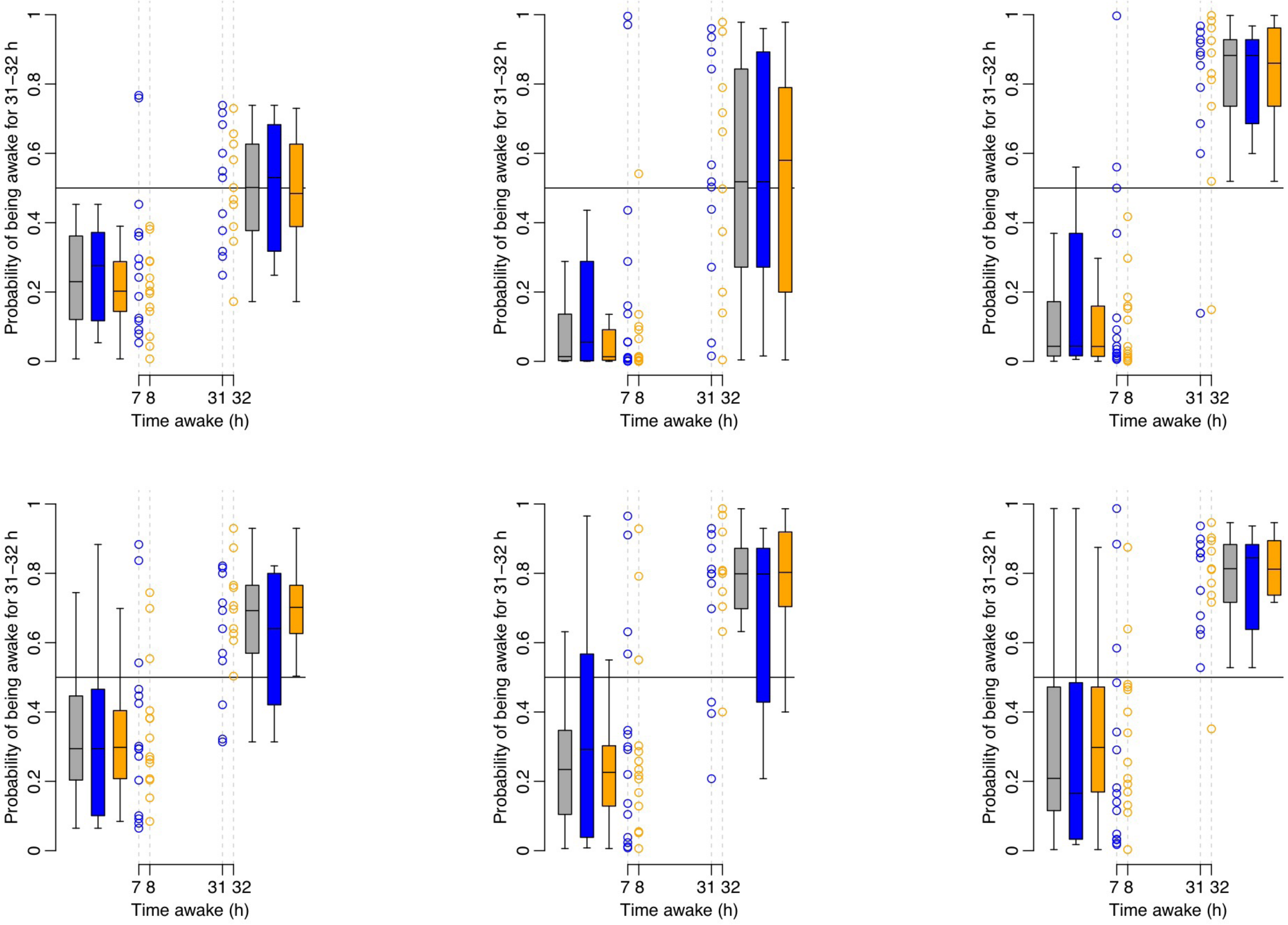

Acute sleep loss, within-subject

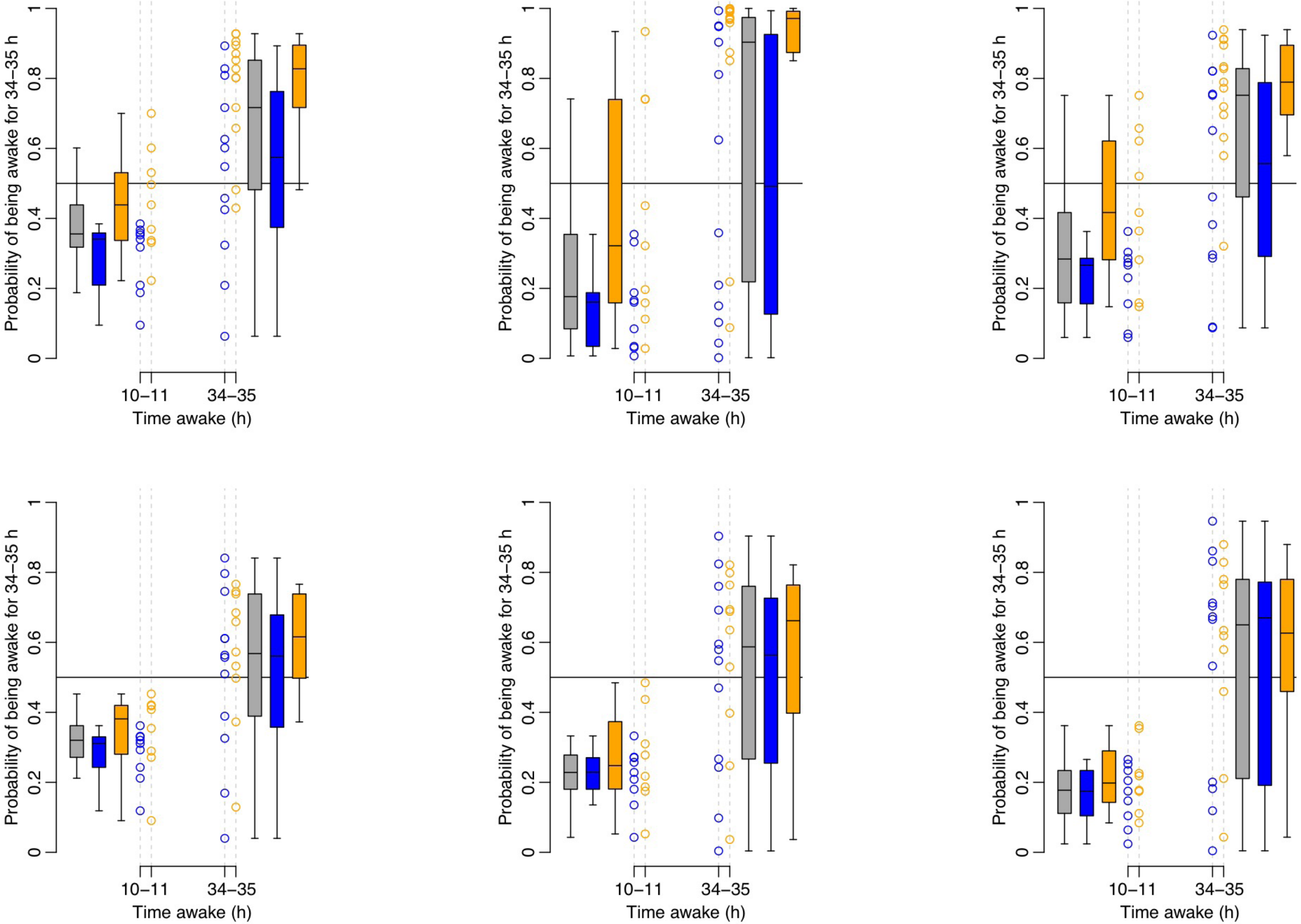

Chronic sleep insufficiency - sample #1 (or #2)

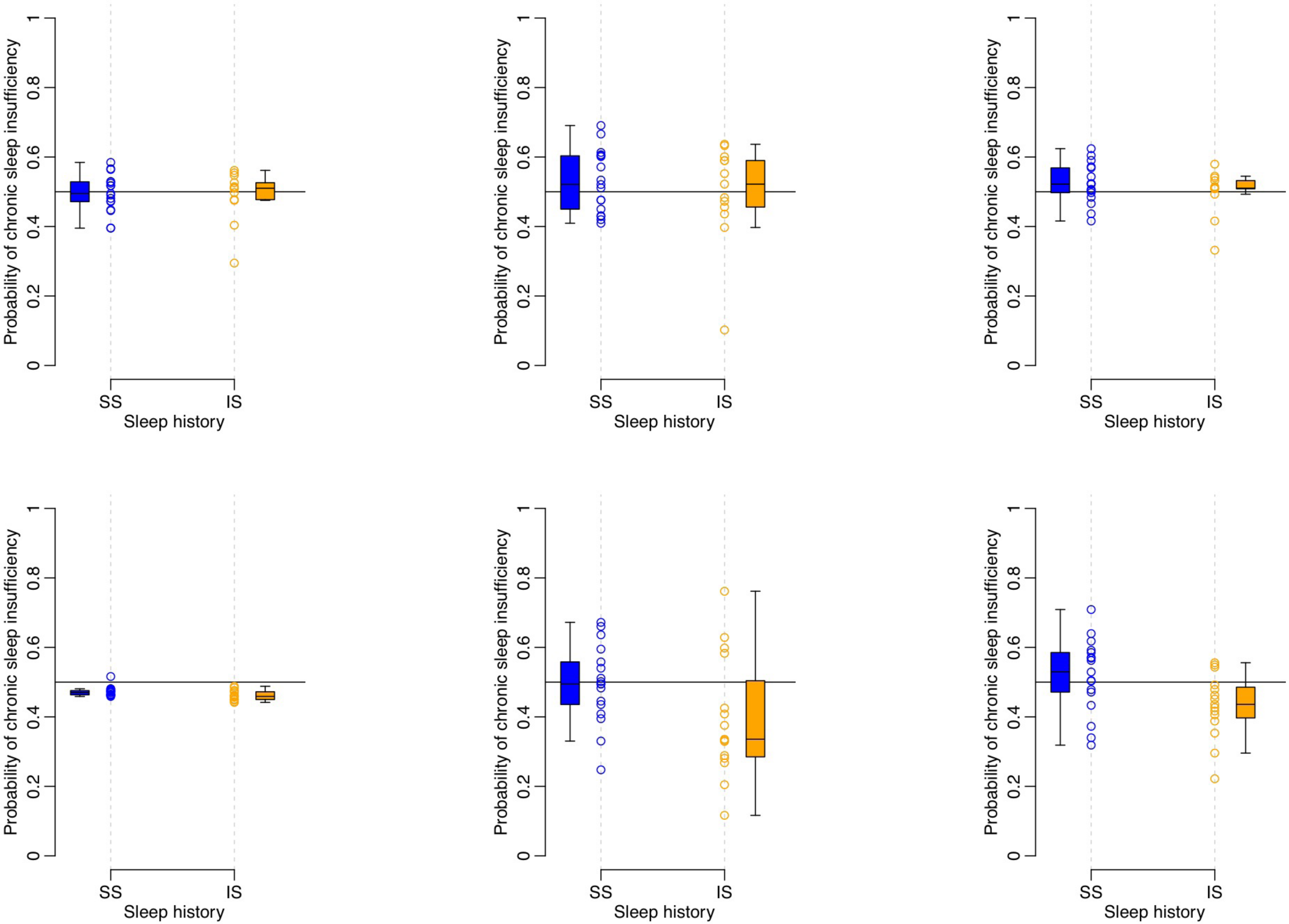

Chronic sleep insufficiency - sample #9 (or #10)

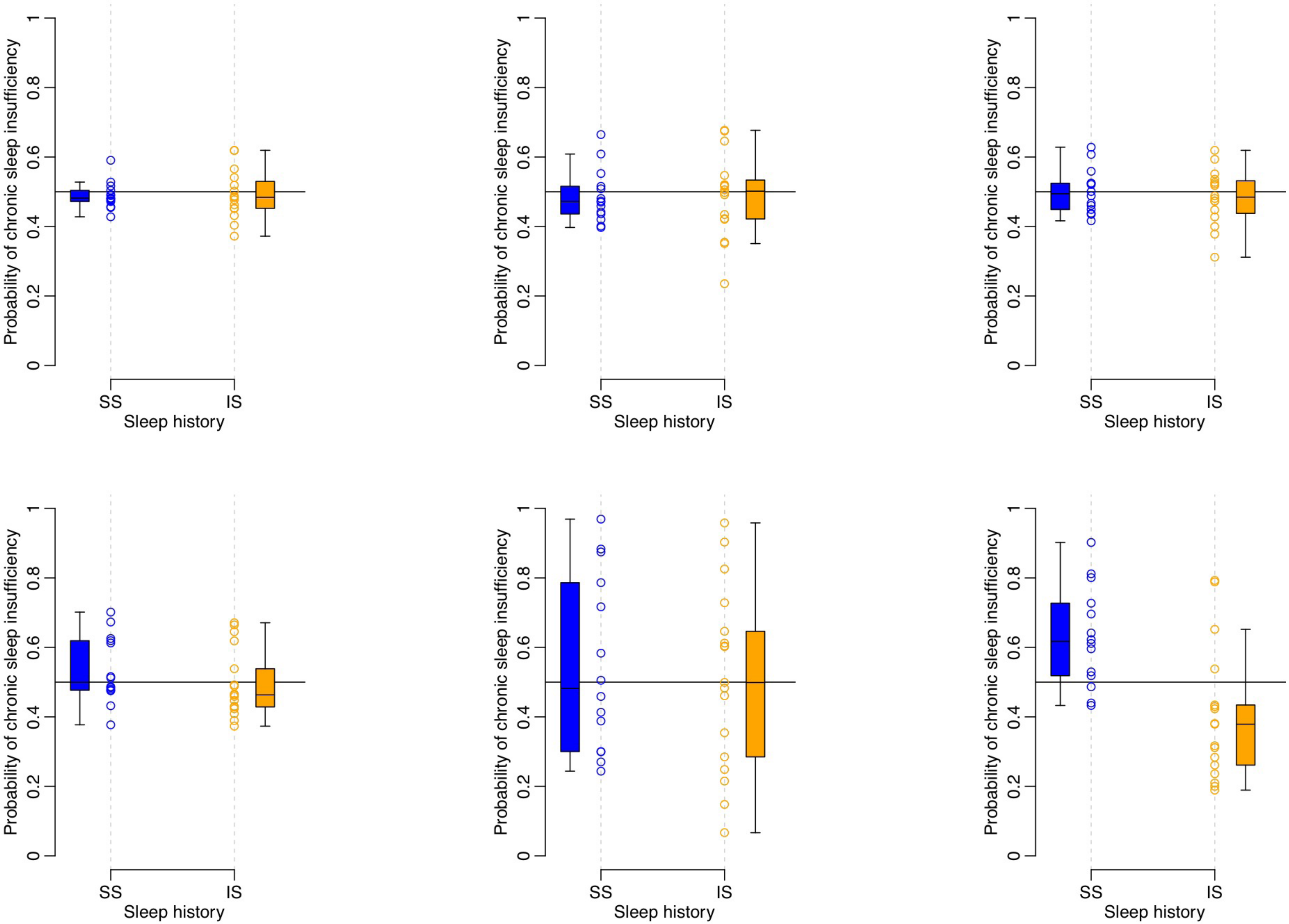

Sleep increase/decrease - sample #1 (or #2)

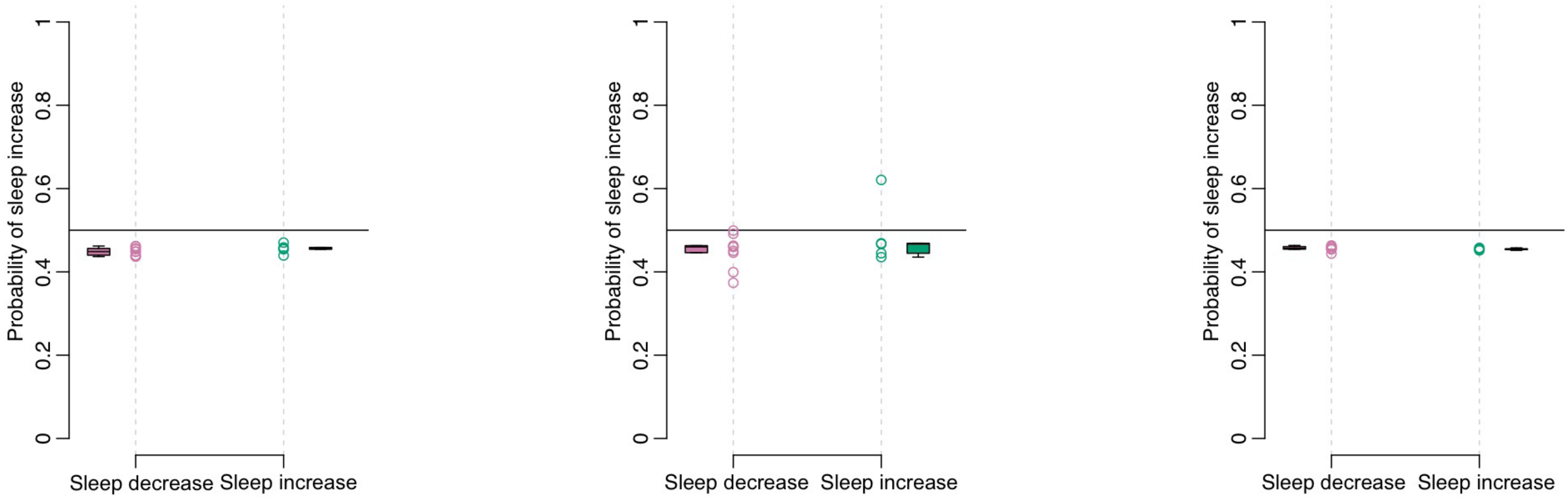

Sleep increase/decrease - sample #2 (or #3)

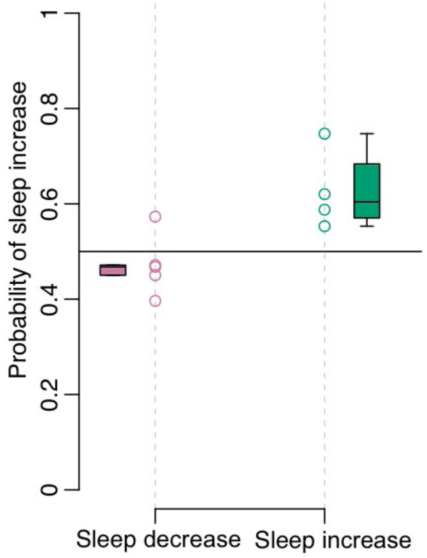

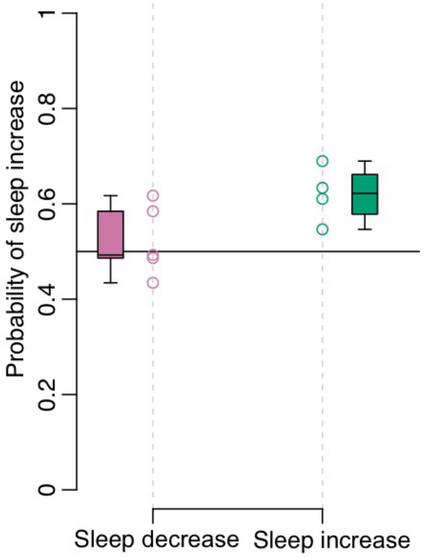

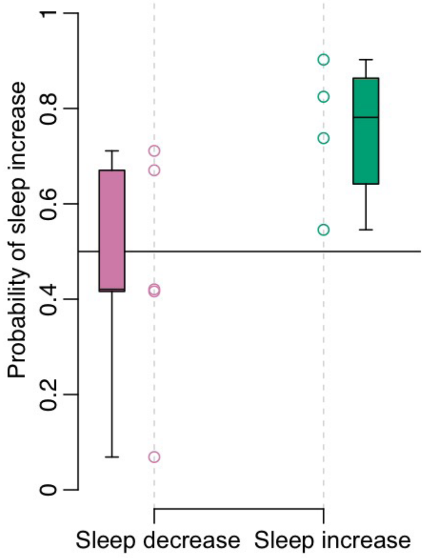

Sleep increase/decrease - sample #3 (or #4)

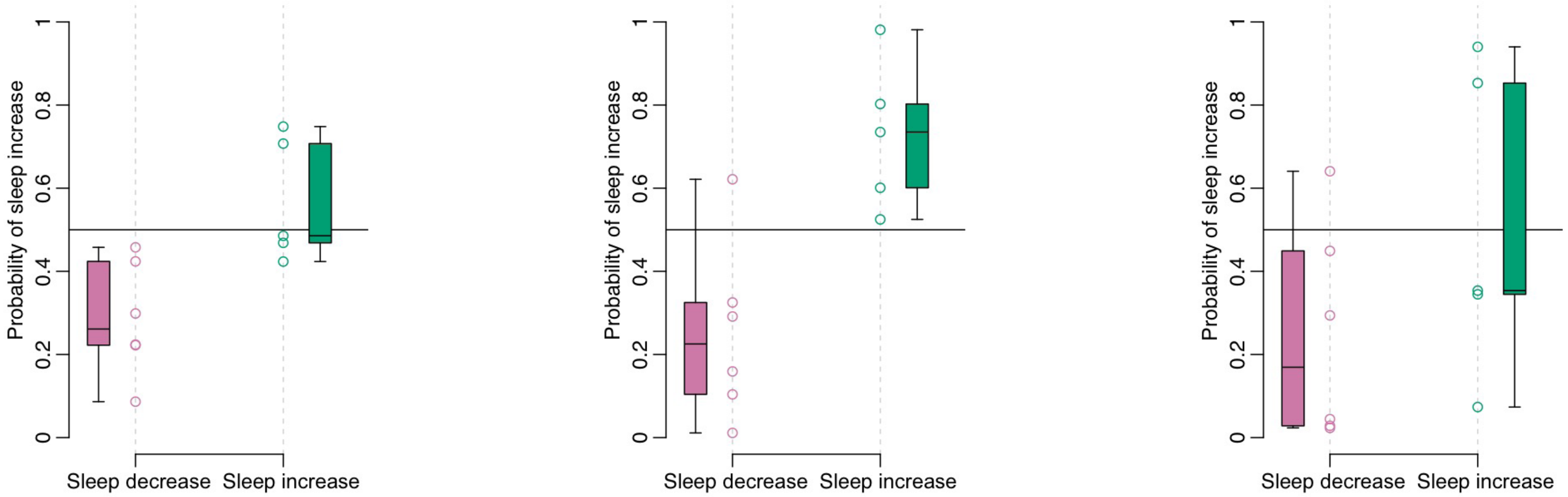

Sleep increase/decrease - sample #4 (or #5)

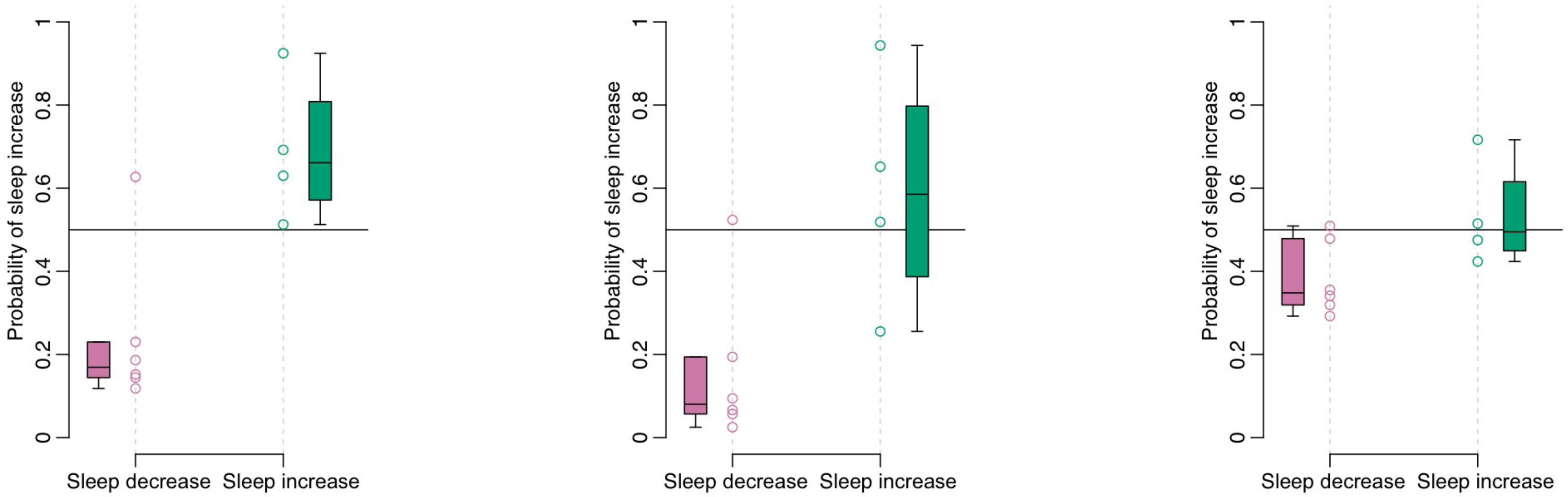

Sleep increase/decrease - sample #5 (or #6)

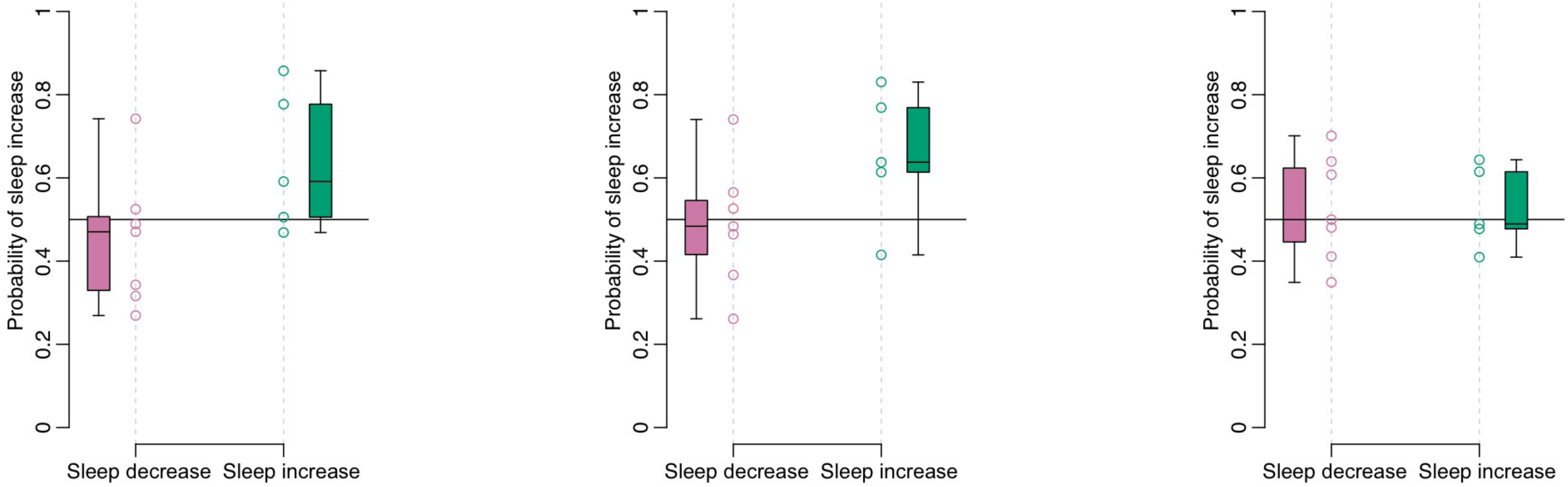

Sleep increase/decrease - sample #6 (or #7)

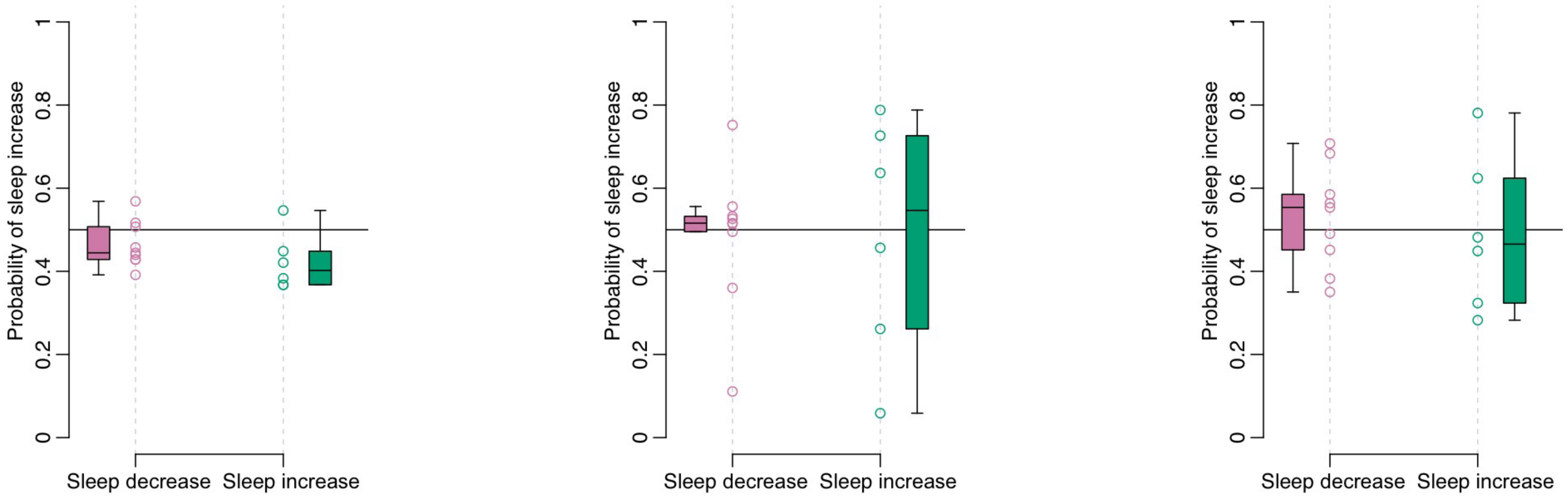

Sleep increase/decrease - sample #7 (or #8)

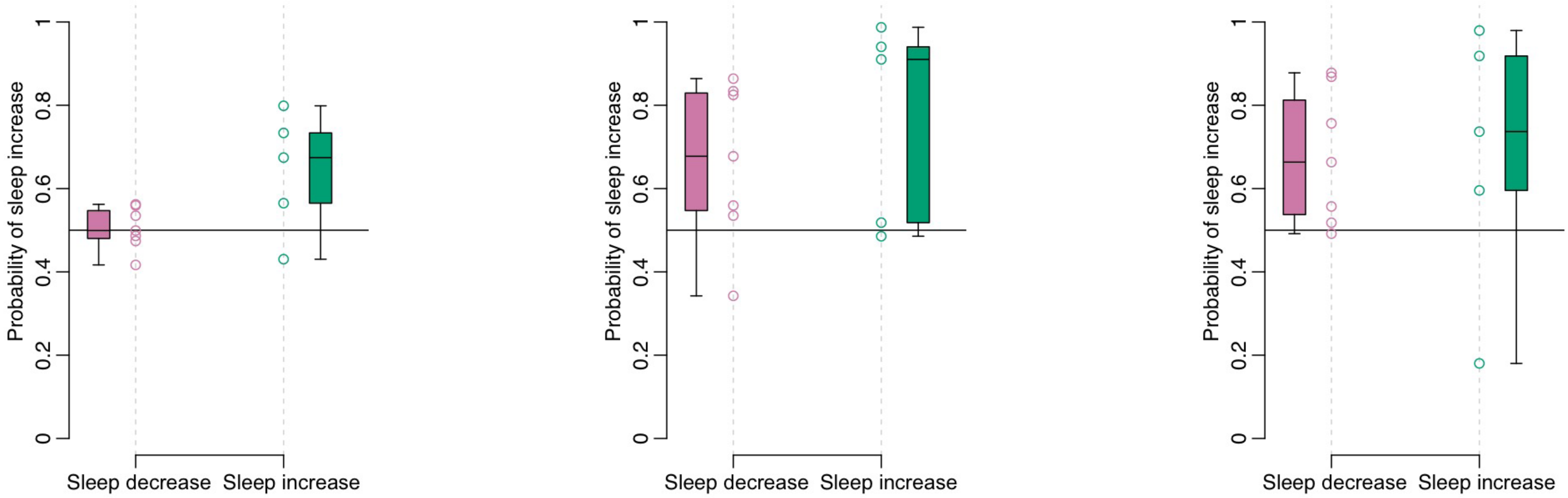

Sleep increase/decrease - sample #8 (or #9)

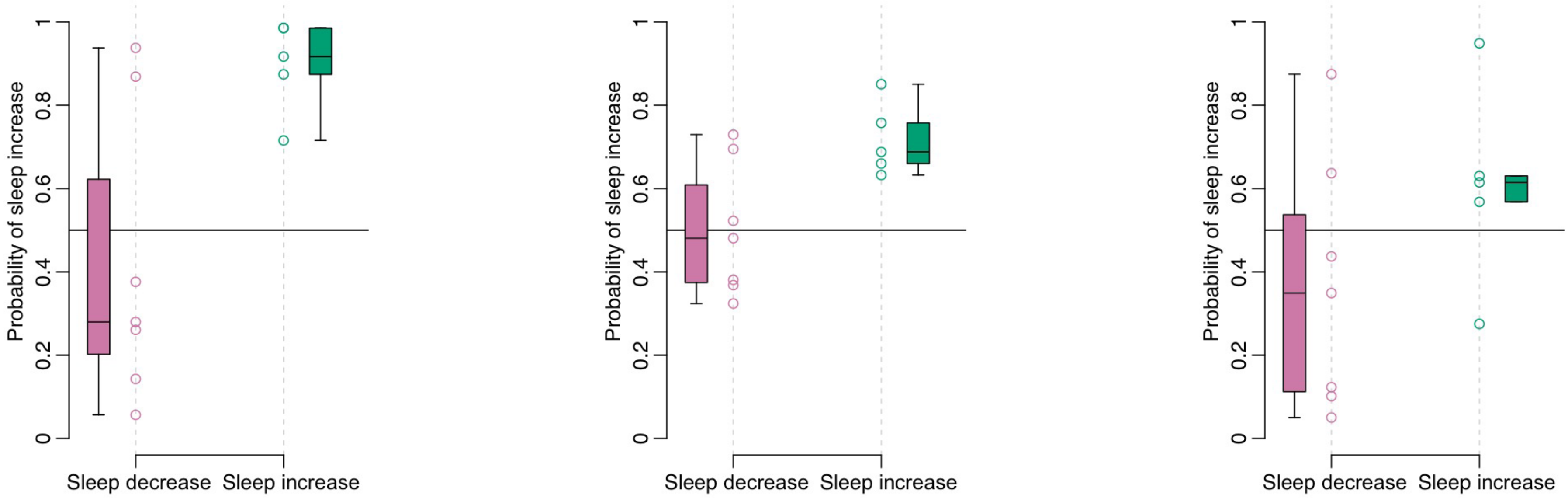

Sleep increase/decrease - sample #9 (or #10)

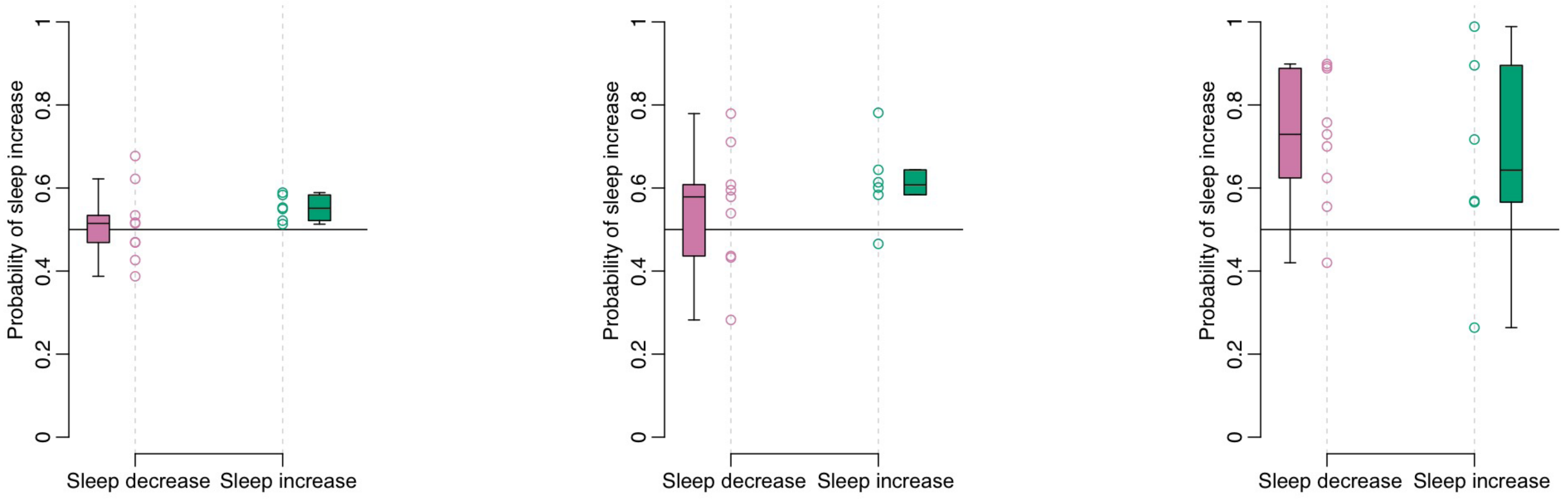

Sleep increase/decrease - sample #10 (or #9)

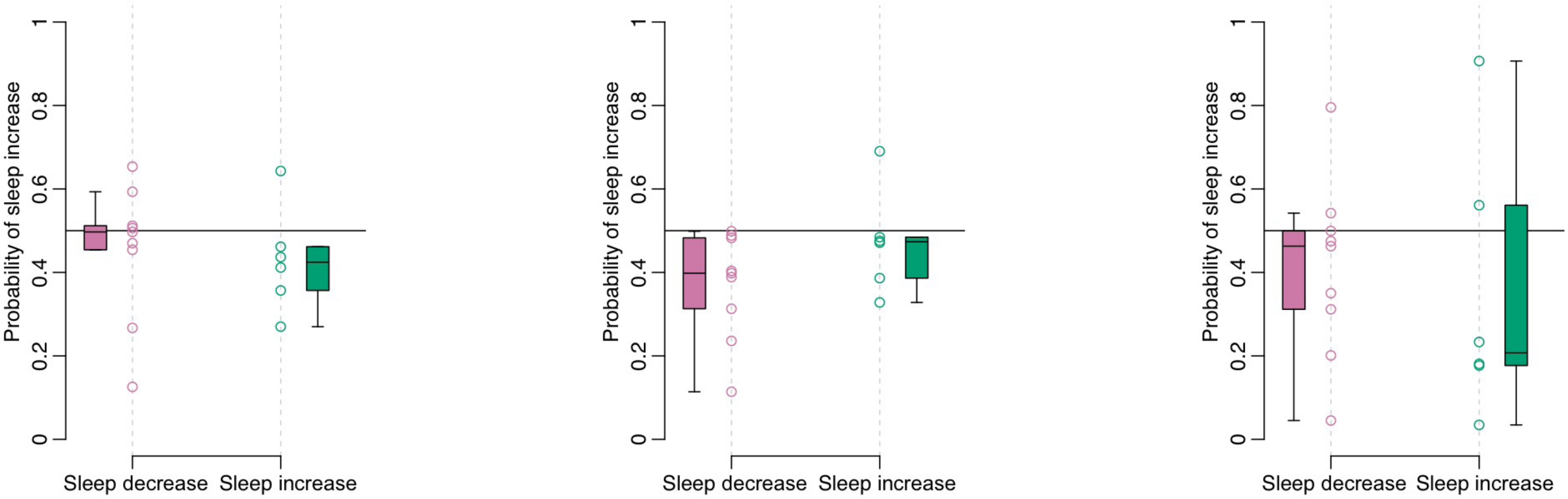

Supplement: zsy186_suppl_Supplementary_File_S1 [file zsy186_suppl_supplementary_file_s1.pdf]
