# Supplementary material for: Identifying and validating blood mRNA biomarkers for acute and chronic insufficient sleep in humans: a machine learning approach
Source: Sleep. 2018 Sep 24;42(1):zsy186. doi: 10.1093/sleep/zsy186 (PMC6335875; doi:10.1093/sleep/zsy186)

'Acute sleep loss, between-subject'

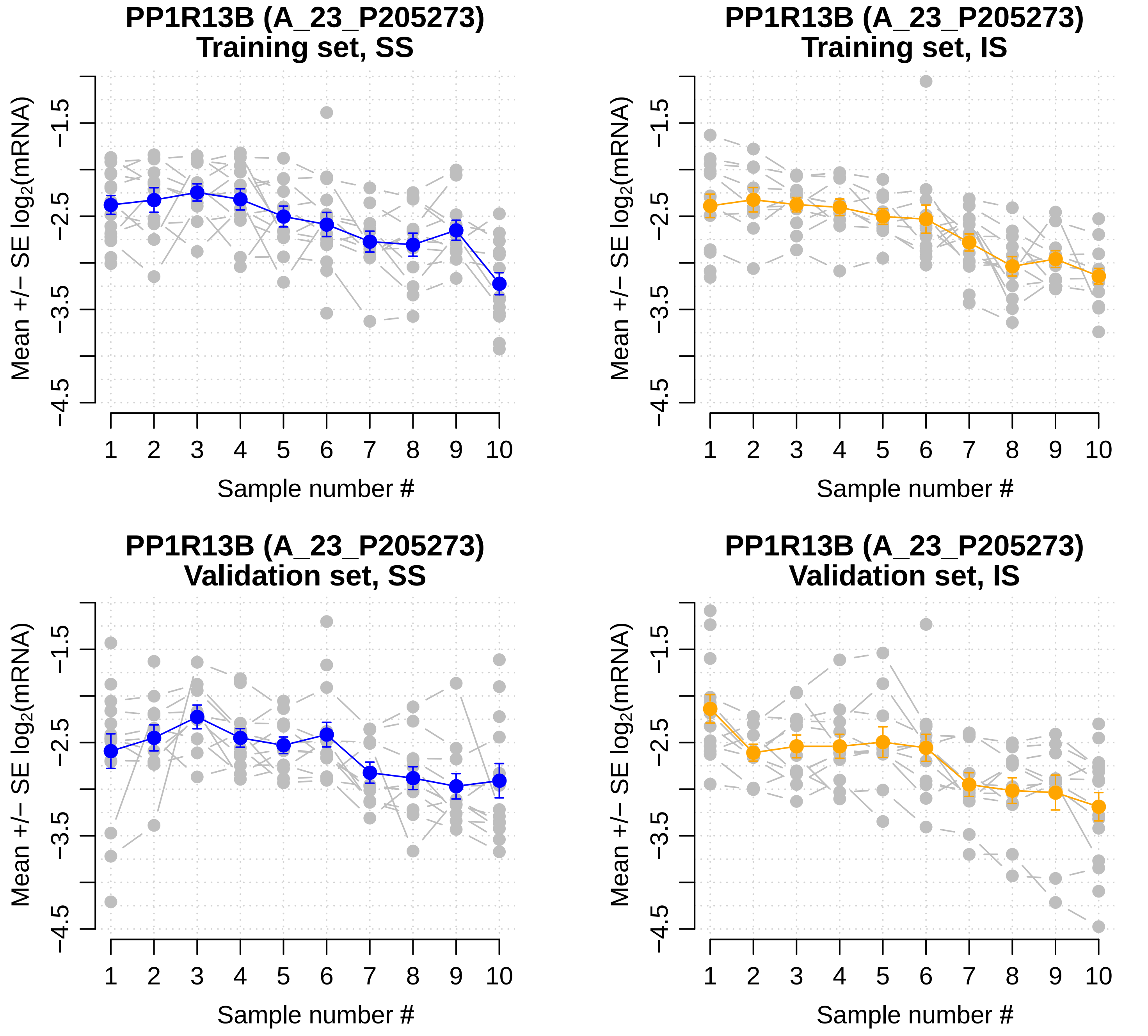

'Sleep increase/decrease'

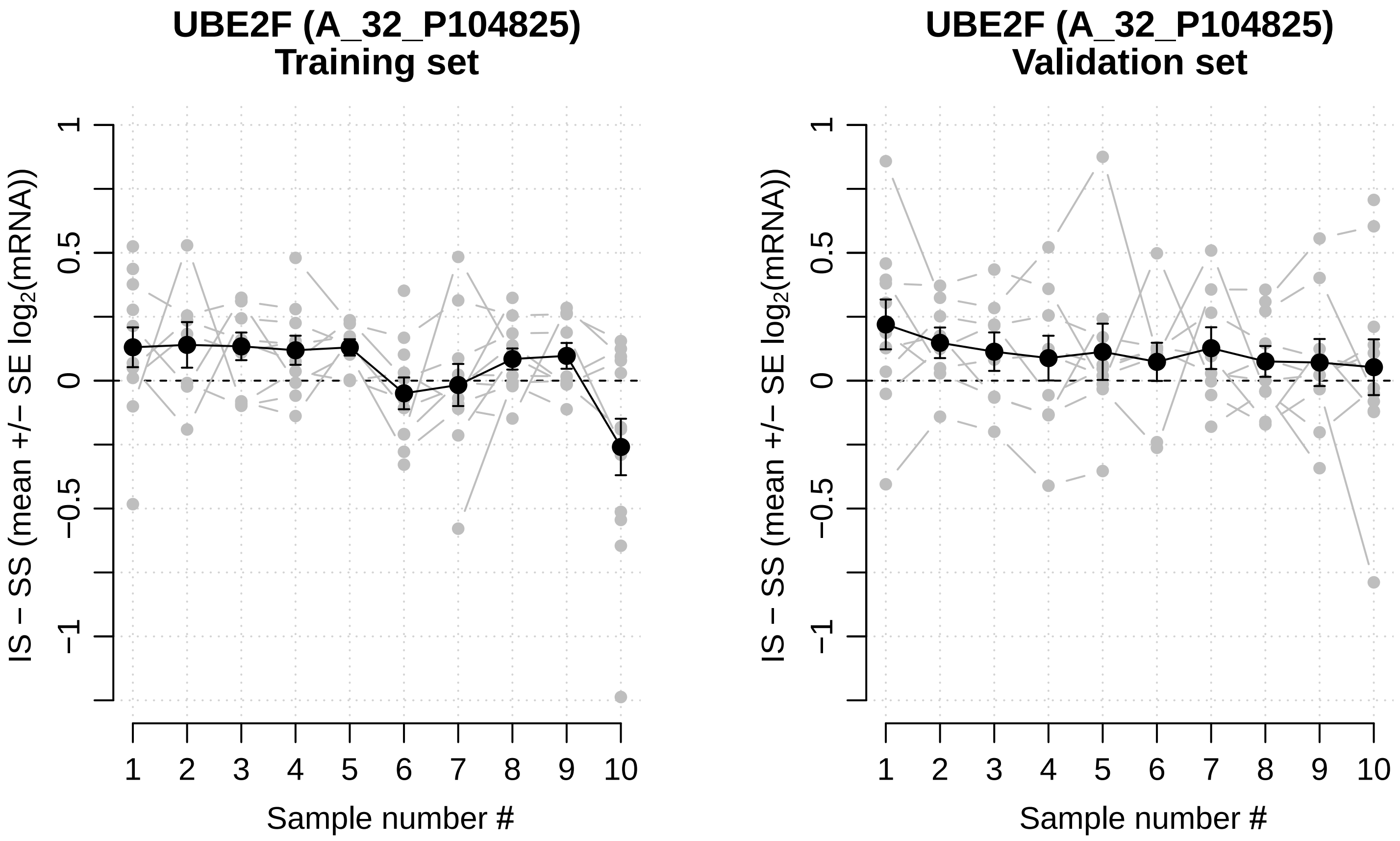

Supplement: zsy186_suppl_Supplementary_File_S2 [file zsy186_suppl_supplementary_file_s2.pdf]
