# Supplementary material for: Identifying and validating blood mRNA biomarkers for acute and chronic insufficient sleep in humans: a machine learning approach
Source: Sleep. 2018 Sep 24;42(1):zsy186. doi: 10.1093/sleep/zsy186 (PMC6335875; doi:10.1093/sleep/zsy186)
Supplement: zsy186_suppl_Supplementary_Legends [file zsy186_suppl_supplementary_legends.docx]

Supplementary material

**Table S1 (.doc): Size and performance of biomarker panels for the prediction/classification of different sleep debt variables.** Data shown for models based on ‘Overlapping participants and samples’ training and validation sets only. ACC = Classification accuracy, Sn = Sensitivity, Sp = Specificity, MCC = Matthew’s Correlation Coefficient.

**File S1 (.pdf): Performance of biomarker panels for sleep debt related variables.** Performance plots for all biomarker panels not shown in the main paper. This includes the *a priori* knowledge based models trained and validated using ‘Unique participants and unique samples’ and all models trained and validated using ‘Overlapping participants and unique samples’, and all sampling points for ‘Sleep increase/decrease’. For classification the decision boundary for classifying samples was set at probability of 0.5.

**File S2 (.pdf): Example mRNA abundance profiles of biomarkers** **for ‘Acute sleep loss, between-subject’ and ‘Sleep increase/decrease’**. All profiles based on samples within the ‘Unique participants and unique samples’ training and validation sets. Grey: participant-specific mRNA abundance profiles. Blue: Mean (across all participants within the training/validation set) profile for the condition of ‘sufficient sleep’. Gold: Mean mRNA abundance profile for the condition of ‘insufficient sleep’. Black: Mean mRNA abundance profile for all differential (insufficient sleep – sufficient sleep) samples.

**Figure S1 (.png): Relationship between biomarker panel size and model performance when classifying ‘Sleep increase/decrease’.** Features are those selected when applying penalized regression (Ridge or Elastic-net) to *a priori* selected features and ‘All features’ as indicated. Data based on the ‘Unique participants and unique samples’ training and validation sets comprising samples #2 or (#3).

**Figure S2 (.png): Distribution of mRNA abundance values for features selected as classifiers of ‘Sleep increase/decrease’.** Features are those selected when applying Elastic-net to ‘All features’ within the ‘Unique participants and unique samples’ training set (without baseline correction) comprising samples #2 (or #3). mRNA abundance data is shown for all samples #2 (or #3) within the ‘Unique participants and unique samples’ training and validation sets, with no baseline correction.

**Data set S1 (.xlsx): Panels of Biomarkers.** Excel workbook where each worksheet contains the list of biomarkers and associated genes for each of the sleep debt variables and approaches discussed.

Data set S2 (.xlsx): Comparison of gene lists associated with different panels of biomarkers. Each column represents a gene list identifiable by the column header in the format of Data split_Sleep debt variable_Input set of features_Regularized regression method. Each row represents a gene. Rows without a gene symbol are identified by the Agilent probe name. 1 indicates presence within the list, 0 absence. Gene annotation as taken from Metascape^37^.

**Figure S3 (.png): Molecular interaction network associated with a priori selected ‘sleep sufficiency’ features.** Molecular interactions as defined by STRING (default settings, confidence score >=0.4). Nodes (molecules) without interactions were removed from the network figure. Node size corresponds to the connectivity (number of edges) of a node within the network.

Figure S4 (.png): Molecular interaction network of genes associated with combined panels for acute and chronic sleep loss. The combined panels for acute sleep loss comprises genes associated with features selected by Elastic-net for predicting/classifying the variables ‘Time awake, between-subject’, ‘Time awake, within-subject’, ‘Acute sleep loss, between-subject’, ‘Acute sleep loss, within-subject’ using the relevant ‘Unique participants and unique samples’ training sets. The combined panels for chronic sleep loss comprises genes associated with features selected by Elastic-net for classifying the variables ‘Chronic sleep insufficiency’ using sample #1 (or #2) and sample #9 (or #10) and ‘Sleep increase/decrease’ using samples #2 (or #3) and sample #9 (or #10) using the relevant ‘Unique participants and unique samples’ training sets. Node size corresponds to the connectivity (number of edges) of a node within the network.

**Data set S3 (.xlsx): Comparison of gene lists associated with different panels of biomarkers for sleep and circadian processes.** Each column represents a gene list. Gene lists from this study are identifiable by the column header in the format of: Data split_Sleep debt variable_Input set of features_Regularized regression method. Additional columns, relating to the circadian phase marker genes reported in ^17^, are identifiable by the column header in the format of: Number of input transcriptome samples_CircadianPhaseMarker_Laing_etal_2017, and Zeitzeiger_deployedLaing_etal_2017, which refers to the deployment of the algorithm Zeitzeiger used to identify circadian phase markers in ^17^. The phase markers identified by Zeitzeiger in ^44^ are provided in column Zeitzeiger_Hughey_etal_2016. Each row represents a gene. Rows without a gene symbol are identified by the Agilent probe name. Note that genes only identified in ^17^ or ^44^ have been removed from the table. 1 indicates presence within the list, 0 absence.
